# Botulinum Toxin in Children and Adolescents—A Comprehensive Review of Clinical Applications

**DOI:** 10.3390/toxins18090360

**Published:** 2026-08-23

**Authors:** Marko Bašković, Vedrana Nikić Čižmek, Jana Buzuk, Bianka Dujić, Danijela Jurić, Kristina Jurković, Karla Pehar, Sara Vuković

**Affiliations:** 1 School of Medicine, Catholic University of Croatia, Ilica 242, 10000 Zagreb, Croatia; 2 Department of Pediatric Surgery, Children’s Hospital Zagreb, Ulica Vjekoslava Klaića 16, 10000 Zagreb, Croatia; 3 School of Medicine, University of Zagreb, Šalata 3, 10000 Zagreb, Croatia; 4 Scientific Centre of Excellence for Reproductive and Regenerative Medicine, School of Medicine, University of Zagreb, Šalata 3, 10000 Zagreb, Croatia; 5 Croatian Academy of Medical Sciences, Kaptol 15, 10000 Zagreb, Croatia; 6 General Hospital “Dr. Ivo Pedišić”, Ulica Josipa Jurja Strossmayera 59, 44000 Sisak, Croatia

**Keywords:** botulinum toxin, children, adolescents, cerebral palsy, spasticity, neurogenic bladder, sialorrhoea, achalasia, paediatric surgery

## Abstract

Botulinum toxin, once known only as the cause of botulism, has become a versatile therapeutic agent whose use in children now extends across many medical and surgical specialties. This narrative review synthesises the clinical use of botulinum toxin in patients up to eighteen years of age. We describe its pharmacology, the non-interchangeable commercial preparations, and the weight-based dosing and safety principles specific to childhood, then examine its applications by organ system. Gastrointestinal uses include functional constipation and internal anal sphincter achalasia, persistent obstruction after surgery for Hirschsprung disease, anal fissure, and achalasia. Urological uses centre on neurogenic and idiopathic detrusor overactivity and on sphincter-directed injection for dysfunctional voiding. Neurological uses are dominated by cerebral palsy spasticity and, on considerably weaker evidence, dystonia, while head and neck uses include sialorrhoea, congenital muscular torticollis, and strabismus. We also address neonatal brachial plexus palsy together with hyperhidrosis and aesthetic use in adolescents. The evidence supporting these uses is markedly uneven. Randomised controlled trials exist for only a small number of paediatric indications, chiefly spasticity in cerebral palsy, neurogenic detrusor overactivity, and chronic sialorrhoea, whereas most remaining applications rest on observational cohorts, small case series, or extrapolation from adult practice, and we grade the certainty of evidence separately for every indication. Only a few indications are formally approved, and most remain off-label, though increasingly supported. Botulinum toxin is a reversible and generally safe adjunct, but rigorous paediatric trials are still needed.

## 1. Introduction

Botulinum toxin is a family of protein neurotoxins produced by the anaerobic, spore-forming bacterium Clostridium botulinum, an organism long feared as the cause of the paralytic illness botulism. The bacterium was first isolated at the end of the nineteenth century by Emile Pierre van Ermengem, who traced a fatal outbreak of food poisoning in the Belgian village of Ellezelles to a toxin-producing bacillus [1]. Decades earlier, the German physician and poet Justinus Kerner had described the clinical picture of sausage poisoning in meticulous detail and had already speculated that the same poison, given in minute amounts, might one day serve to treat disease [2]. That remarkable prediction was realised in the early 1970s, when Alan B. Scott adapted purified type A toxin as a nonsurgical means of weakening the extraocular muscles, and in 1980 he reported the first controlled human series in strabismus, transforming a feared poison into a precise therapeutic tool [3,4].

The therapeutic action of botulinum toxin rests on an exquisitely selective molecular mechanism. The heavy chain of the toxin binds to receptors on cholinergic nerve terminals, the molecule is internalised, and the catalytic light chain then cleaves proteins of the soluble N-ethylmaleimide-sensitive factor attachment protein receptor complex, thereby blocking the calcium-dependent release of acetylcholine at the neuromuscular junction and at autonomic synapses. Type A toxin, the serotype used most widely in clinical practice, cleaves the synaptosomal-associated protein of 25 kilodaltons, producing a focal and reversible chemodenervation that persists for several months until axonal sprouting and the turnover of nerve terminals restore normal transmission. Seven classical serotypes, designated A through G, share this general strategy but differ in their intracellular targets, potency, and duration of effect, a diversity that explains why the various commercial preparations are not interchangeable and why doses expressed in units of one product cannot be transferred to another [5]. This mechanism, however, explains far less about clinical outcome than it might appear to. The same presynaptic block is delivered whether the target is a spastic gastrocnemius, an overactive detrusor, or a salivary gland, yet the evidence supporting these uses differs enormously in quality. What varies between indications is not the pharmacology but the accessibility and size of the target tissue, the choice of outcome measure, the feasibility of blinding and of placebo control in children, and the rigour of the studies actually performed. For that reason the sections that follow are organised by organ system but are read most usefully alongside the strength of evidence behind each indication, which we state explicitly throughout.

After its safety in humans had been established, the clinical reach of botulinum toxin widened rapidly, moving from ophthalmology into neurology, urology, gastroenterology, otolaryngology, and aesthetic medicine, a breadth already catalogued in a landmark review at the start of the 1990s [5,6,7]. In the paediatric field, the decisive moment came in 1993, when Koman et al. reported the first use of intramuscular type A toxin to manage the dynamic muscular imbalance of cerebral palsy, an observation that inaugurated the modern era of chemodenervation in children [8]. Randomised and controlled trials soon followed, and an evidence-based assessment by the American Academy of Neurology concluded that focal spasticity in children could be reduced safely and reproducibly, so that cerebral palsy, a disorder that affects roughly two of every thousand live births, has remained the single most frequent paediatric indication worldwide [9,10].

Treating children is not simply a matter of transposing adult practice to a smaller body. The immature and still developing neuromuscular system, the demands of ongoing skeletal growth, dosing that must be calculated by body weight and capped to limit systemic spread, the frequent need for procedural sedation or general anaesthesia, and the largely off-label regulatory status of most indications together set the paediatric setting apart from adult use. At present, the formally approved paediatric indications remain limited and include blepharospasm, strabismus, lower limb and focal spasticity associated with cerebral palsy, and neurogenic detrusor overactivity, whereas a large and expanding range of gastrointestinal, urological, glandular, and musculoskeletal conditions are treated off-label on the strength of accumulating but frequently low-certainty evidence [6].

Against this background, the present narrative review sets out to provide a comprehensive and methodologically transparent synthesis of the clinical use of botulinum toxin in patients up to eighteen years of age. We examine gastrointestinal, urological, neurological and neuromuscular, head and neck, and peripheral nerve applications, together with aesthetic use and the pharmacological and safety considerations that are specific to childhood. We aim to give the practising clinician and the researcher a single, detailed, and critically appraised reference source, and to identify the areas in which the evidence remains thin and where future study is most needed.

## 2. Materials and Methods

This work was conducted as a narrative review enriched by a structured and reproducible literature search so that the breadth characteristic of the narrative format could be combined with methodological transparency. The review considered the use of botulinum toxin in human patients from birth up to and including the age of eighteen years, across every clinical discipline in which the agent is applied.

### 2.1. Search Strategy and Information Sources

We interrogated the electronic databases PubMed and MEDLINE, Scopus, Web of Science, Embase, and the Cochrane Central Register of Controlled Trials from their inception through 30 June 2026. The core search combined the controlled vocabulary and free text terms botulinum toxin, botulinum neurotoxin, onabotulinumtoxinA, abobotulinumtoxinA, incobotulinumtoxinA, and rimabotulinumtoxinB with the paediatric filters child, children, infant, newborn, neonate, adolescent, and paediatric, and with terms for each clinical indication considered in this review.

To maximise sensitivity, the indication-specific terms included constipation, faecal incontinence, anal fissure, achalasia, overactive bladder, neurogenic detrusor overactivity, detrusor sphincter dyssynergia, posterior urethral valve, myelomeningocele, cerebral palsy, spasticity, dystonia, sialorrhoea, drooling, congenital muscular torticollis, obstetric brachial plexus palsy, strabismus, hyperhidrosis, and aesthetic or cosmetic use, among others. The reference lists of all retrieved articles and of relevant systematic reviews were screened by hand to identify additional records not captured by the electronic search. Eligibility was restricted to reports published in English.

### 2.2. Eligibility Criteria, Study Selection, and Data Synthesis

Studies were eligible when they reported the clinical use of any botulinum toxin preparation in patients up to eighteen years of age and provided extractable information on indication, dosing, technique, efficacy, or safety. Randomised controlled trials, non-randomised comparative studies, prospective and retrospective cohorts, case series, and authoritative clinical guidelines were all considered, reflecting the reality that high-level evidence is scarce for many paediatric indications. Reports confined to adults, purely preclinical studies without clinical correlation, and conference abstracts without full data were excluded. A small number of cited articles could not be retrieved in full text. We retained them, because several are historically important and appear in every account of this field, but we appraised none of them, and any design detail given for such a study is taken from its published abstract and is identified as such at the point of use. Because the aim was a qualitative synthesis rather than a meta-analysis, findings were summarised narratively and organised by organ system and indication, with quantitative results reported as presented in the primary sources.

Two points about the method should be stated plainly, because they define the standard against which this work should be judged. This is a narrative review supported by a structured search. It is not a systematic review, it was not registered, and it does not follow the PRISMA reporting framework. We therefore did not carry out duplicate independent screening with formal adjudication of disagreements, and we do not report record counts at each stage of retrieval and screening because those are the instruments of a systematic review, and reporting them here would imply a design we did not follow. What we do report is the eligibility criteria, an appraisal of the studies on which our conclusions actually rest, and an explicit certainty rating for every indication.

In preparing and appraising the manuscript, we used the six domains of the Scale for the Assessment of Narrative Review Articles as a self-assessment checklist, applying its standards for the justification, the aims, the literature search, the referencing, and the presentation of evidence in a narrative review [11].

The Scale for the Assessment of Narrative Review Articles appraises the reporting quality of this review itself and says nothing about the methodological quality of the primary studies cited within it. We therefore appraised, separately and explicitly, the studies on which the certainty rating of each indication depends, rather than every reference cited in the manuscript. This is the proportionate approach for a narrative synthesis, since a substantial part of our reference list consists of historical, pharmacological, regulatory, and guideline sources for which a clinical risk-of-bias instrument is not meaningful. Randomised controlled trials were assessed with the revised Cochrane risk-of-bias tool, non-randomised comparative studies and cohorts with the ROBINS-I tool, and case series and uncontrolled reports with the Joanna Briggs Institute critical appraisal checklist for case series. No study was excluded on the basis of its appraisal, because applying a minimum quality threshold would have eliminated the entire evidence base for several indications and would have given a misleadingly narrow picture of current practice. The appraisal of each of these studies is presented in Supplementary Table S1 and is carried forward into the certainty rating assigned to each indication in Table 3, so that the reader can see directly which conclusions rest on robust designs and which do not. Two further points follow from this scope. Systematic reviews and meta-analyses cited as evidence were appraised with AMSTAR-2 rather than with an instrument designed for primary studies. Clinical practice guidelines, evidence-based assessments, and consensus statements are cited in this review as sources of recommendation rather than as primary evidence, and they were therefore not appraised with any study-level instrument.

### 2.3. Grading the Certainty of the Evidence

Because this review spans indications whose evidence bases differ by orders of magnitude, we applied one explicit grading scheme throughout, adapted from the GRADE framework and simplified for a narrative synthesis. Evidence was rated high where two or more adequately powered randomised controlled trials in children reported consistent results, moderate where a single randomised trial or several concordant prospective controlled studies were available, low where the evidence came only from observational cohorts or comparative studies without randomisation, and very low where it rested on small case series, uncontrolled reports, expert consensus, or extrapolation from adult data. Every indication discussed in Section 4, Section 5, Section 6, Section 7, Section 8 and Section 9 carries one of these four ratings, and all ratings are collected in Table 3. Two indications carry an intermediate rating of low to moderate, because their evidence base straddles two of the four levels, and one indication carries a split rating because the certainty differs materially between its clinical subgroups. We have recorded these as they are rather than force them into a single category, and in each case the reason is stated in the accompanying clinical position paragraph. The basis for each rating, that is, the supporting references, the predominant study design behind them, the risk-of-bias profile of those studies, and the proportion of the supporting studies that were formally appraised, is set out in Supplementary Table S2. Where a treatment effect is described, we also state whether the outcome measured was a physiological variable such as muscle tone, sphincter pressure, or bladder capacity, or a functional outcome at the level of activity or participation, because these are not equivalent, and the distinction is developed further at the start of Section 6.

## 3. Pharmacology, Formulations, and Principles of Use in Children

A rational use of botulinum toxin in children depends on a clear grasp of how the molecule acts, of the differences between the commercial preparations, and of the dosing and safety principles that are specific to the growing body. These pharmacological foundations are summarised here because they underpin every indication discussed in the sections that follow.

### 3.1. Mechanism of Action and Duration of Effect

The clinical effect of every licenced preparation derives from the same sequence of events at the cholinergic nerve terminal. The heavy chain of the toxin first binds to a polysialoganglioside and to a protein receptor on the presynaptic membrane; the molecule is taken up by receptor-mediated endocytosis, the light chain is translocated across the endosomal membrane into the cytosol, and there it acts as a zinc-dependent protease that cleaves a component of the fusion machinery that docks acetylcholine vesicles to the membrane. Type A and type E toxins cleave the synaptosomal-associated protein of 25 kilodaltons, whereas type B, D, F, and G toxins cleave the vesicle-associated membrane protein, and each cut halts the calcium-triggered release of acetylcholine so that the target muscle or gland is functionally denervated without structural injury to the nerve. Because the block is presynaptic and the axon itself remains intact, the effect is temporary. New nerve sprouts and the eventual recovery of the original terminal restore transmission over roughly three to four months, which is why treatment must be repeated and why the interval between injections is a central practical concern in children who may require therapy for many years [5].

The molecular detail behind this sequence has been established over several decades. Crystalline type A toxin suitable for human use was first produced by Schantz et al., whose purification methods underlie the products still in use today [12]. The nature of the intracellular target became clear when Schiavo et al. showed that tetanus and type B botulinum toxins are zinc-dependent proteases that cleave synaptobrevin, revealing the enzymatic basis of the block [13]. The remarkable neurospecificity of the molecule was later explained by a double receptor model, in which the heavy chain binds first to a polysialoganglioside on the presynaptic membrane and then to a protein exposed on the lumen of a synaptic vesicle during transmitter release, type A toxin using synaptic vesicle glycoprotein 2 for this second step [14,15,16]. Because this uptake depends on vesicle recycling, more active nerve terminals internalise more toxin, which helps to explain why more active muscles tend to respond more strongly. Recovery then follows a characteristic biphasic course, transmission being restored first through a transient network of nerve terminal sprouts and later returning to the original ending as those sprouts regress [17].

Two limits of this mechanistic account deserve emphasis because mechanism is often invoked to justify clinical practice in indications where clinical data are thin. The first is that the mechanism does explain why the duration of effect is broadly similar across targets as different as an equinus gastrocnemius, an overactive detrusor, and a parotid gland, since recovery depends on the turnover of nerve terminals rather than on the tissue injected. The second is that the mechanism explains very little about why individual children respond so differently to the same weight-adjusted dose. Differences in muscle fibre composition and in the density of neuromuscular junctions, in the proportion of dynamic tone to fixed contracture within the target, in injected volume and dilution, in the accuracy of localisation, and in the intensity of the rehabilitation delivered afterwards all shape the observed result, and none of these is a property of the molecule. Mechanistic plausibility should therefore never be read as evidence of clinical benefit for a given indication, and in this review each indication is graded on clinical data alone.

### 3.2. Available Formulations and the Problem of Unit Interchangeability

Several distinct products are marketed, and they are not clinically equivalent. The type A preparations in widest use are onabotulinumtoxinA, abobotulinumtoxinA, and incobotulinumtoxinA, to which newer type A products such as prabotulinumtoxinA, letibotulinumtoxinA, and the long acting daxibotulinumtoxinA have been added, while rimabotulinumtoxinB is the only licenced type B product, as summarised in Table 1 [18]. Although all type A products contain the same 150 kilodalton neurotoxin derived from the Hall strain of Clostridium botulinum, they differ in their complexing proteins, excipients, and manufacturing, and incobotulinumtoxinA is supplied as the purified neurotoxin without accessory proteins [18]. The potency of each product is expressed in mouse units defined by a manufacturer-specific assay, so that a unit of one product is not biologically equivalent to a unit of another and the numerical doses cannot be exchanged. Reported conversion ratios between onabotulinumtoxinA and abobotulinumtoxinA vary widely, commonly lying between about one to two and one to three, and no single factor is valid across all indications, a source of error that is particularly dangerous in small children where the margin between an effective and an excessive dose is narrow [18]. For this reason, the toxin should always be prescribed by its specific nonproprietary name together with the brand, and the dose should never be carried over from one preparation to another [6,18]. Formal analyses of conversion confirm that no fixed ratio applies across all indications, with reported abobotulinumtoxinA to onabotulinumtoxinA ratios of about three to one or lower, and regulatory and manufacturer guidance continues to stress that the products are not interchangeable [19,20]. We state explicitly that the newer type A products carry no paediatric evidence base. The prescribing information for daxibotulinumtoxinA records that studies have not been conducted in patients below eighteen years of age and that safety and effectiveness in this population have not been established, and we identified no published paediatric efficacy or safety data for daxibotulinumtoxinA, prabotulinumtoxinA, or letibotulinumtoxinA in any indication. These products appear in Table 1 only so that the pharmacological landscape is complete, and their inclusion should not be read as implying any established paediatric role.

Because cross-product confusion is a recurrent source of preventable harm rather than a theoretical caveat, we state the point here in the strongest terms. Dose carry-over between preparations is a documented real-world cause of paediatric medication error, and its consequences are asymmetric in children, since an inadvertent threefold overdose in a ten-kilogram child carries a risk of dysphagia, generalised weakness, and respiratory compromise that has no counterpart in an adult receiving the same absolute excess. Every prescription, pharmacy label, reconstitution record, and clinical note should therefore carry the nonproprietary name, the brand name, the total units administered, the volume and concentration after reconstitution, and the dose expressed in units per kilogram of body weight. Where a service holds more than one preparation, the products should be stored separately, a second practitioner should independently verify the calculated dose before reconstitution, and any change in preparation in an individual child should be treated as a new course of treatment with the dose recalculated from first principles rather than converted by any published ratio.

**Table 1:** Botulinum toxin preparations relevant to clinical practice.

Nonproprietary Name	Common Brand Names	Serotype	Distinguishing Feature
onabotulinumtoxinA	Botox, Vistabel	A	Neurotoxin within a protein complex, the first product in clinical use
abobotulinumtoxinA	Dysport, Azzalure	A	Distinct unit scale, numerical doses several times higher than onabotulinumtoxinA
incobotulinumtoxinA	Xeomin, Bocouture	A	Purified neurotoxin free of complexing proteins
prabotulinumtoxinA	Jeuveau, Nabota	A	Type A product, used chiefly for aesthetic indications
letibotulinumtoxinA	Letybo, Botulax	A	Type A product of more recent introduction
daxibotulinumtoxinA	Daxxify	A	Type A product formulated for a longer duration of effect
rimabotulinumtoxinB	Myobloc, Neurobloc	B	The only licenced type B product

Products are listed by their nonproprietary (International Nonproprietary Name) designation. Brand availability and the range of approved indications vary between countries.

### 3.3. Dosing, Injection Technique, and Localisation in Children

Paediatric dosing is calculated by body weight and is constrained by ceilings that are intended to limit the quantity of toxin reaching the systemic circulation. European consensus recommendations for children with cerebral palsy place limits on the dose delivered to an individual muscle, on the dose per injection site, and on the total dose given in a single session, and these weight-based frameworks have become the practical basis for dosing across most paediatric indications even where formal approval is lacking [21,22]. The freeze-dried powder is reconstituted with preservative-free saline shortly before use, and the concentration chosen influences how far the toxin spreads through the tissue, a more dilute solution diffusing more widely and a more concentrated one remaining more focal [18]. Accurate placement matters because the toxin must reach the neuromuscular junction or the target gland, and localisation by anatomical landmarks is increasingly supplemented or replaced by ultrasound guidance, electrical stimulation, or electromyography, particularly for deep or small muscles. Because injection is uncomfortable and young children are rarely able to hold still, procedures are frequently performed under topical or inhaled analgesia, conscious sedation, or brief general anaesthesia, and the choice of setting is itself an important part of planning treatment [22].

A systematic review and meta-analysis has confirmed that pharmacological procedural sedation reduces the pain and distress of injection in children with cerebral palsy, and the choice between topical anaesthesia, conscious sedation, and general anaesthesia is guided by the number of sites, the depth of the target muscles, and the child’s ability to cooperate [23].

### 3.4. Immunogenicity, Distant Spread, and the Boxed Warning

Repeated exposure to a foreign protein carries a risk of immunisation. A minority of patients develop neutralising antibodies to the neurotoxin, and although secondary loss of response is uncommon, it becomes more likely when large doses are given at short intervals, so that using the lowest effective dose and the longest tolerable interval remains the accepted strategy for reducing immunogenicity, a consideration that is especially relevant in children who face years of repeated treatment. The formulation carrying the smallest load of foreign protein might in theory present the lowest antigenic burden, although the clinical significance of the differences between products remains debated [24]. Systemic safety is dominated by the possibility that toxin may spread beyond the injected muscle, and regulatory agencies have placed a boxed warning on all preparations because distant spread can, though rarely, produce symptoms resembling botulism, such as generalised weakness, difficulty swallowing, and, at the extreme, respiratory compromise [6]. The great majority of adverse events reported in children have been mild and local, consisting of transient weakness, pain, or fatigue, whereas the most serious events, including the rare deaths recorded in the literature, have occurred in children with cerebral palsy who received high off-label doses, an observation that underlines the importance of weight-based dosing and conservative total doses in this population [6,25].

## 4. Gastrointestinal Applications

The gastrointestinal tract provides some of the oldest and best-established paediatric applications of botulinum toxin, nearly all of which exploit a single principle, the temporary relaxation of a smooth or striated sphincter that fails to relax as it should. Because the effect is reversible and avoids the permanent weakening that follows surgical division of a sphincter, chemodenervation has become an attractive first step or a diagnostic manoeuvre in several disorders of defecation and swallowing [6].

### 4.1. Functional Constipation and Internal Anal Sphincter Dysfunction

A subgroup of children with severe functional constipation fail to relax the internal anal sphincter during attempted defecation, and in these patients injection of botulinum toxin into the sphincter can restore a more normal pattern of emptying. In a double-blind randomised trial, Keshtgar et al. showed that intrasphincteric toxin improved symptoms and lowered resting anal pressure in children with chronic idiopathic constipation, with benefit sustained on long-term follow-up [26]. Comparable results were reported by Irani et al., who treated children with demonstrable internal anal sphincter dysfunction [27], and by Chumpitazi et al., whose long-term series in children with a nonrelaxing internal anal sphincter documented durable improvement in a substantial proportion of patients after one or more injections [28]. A further randomised study confirmed that injection into the anal sphincter is an effective and safe treatment for refractory constipation in children [29]. When the clinical picture resembles Hirschsprung disease but rectal biopsy shows ganglion cells and manometry shows an absent rectoanal inhibitory reflex, the condition is termed internal anal sphincter achalasia, and a meta-analysis by Friedmacher and Puri found that intrasphincteric toxin produced symptomatic relief, although posterior myectomy achieved a higher rate of durable response, with transient faecal incontinence, nonresponse, and the need for further surgery all more common after toxin [30].

The paediatric experience has broadened considerably since these early reports. Ciamarra et al. characterised internal anal sphincter achalasia in children and confirmed the value of intrasphincteric toxin in this specific group [31], and larger institutional series have since described outcomes across mixed populations of functional constipation, Hirschsprung disease, and anorectal malformation, with symptomatic improvement after a first injection in about seventy per cent of children [32]. This figure derives from one retrospective institutional cohort of mixed diagnoses without a control group, and it should be read as a single-centre estimate rather than as a response rate validated across studies. A series from a neurogastroenterology programme found that the toxin was effective regardless of whether anorectal manometry showed a nonrelaxing sphincter, which calls into question the need for manometric selection before treatment [33]. Delivery has also been refined, and a randomised trial showed that intrarectal administration by electromotive delivery, without a needle, improved intractable constipation in children [34]. A large review of complications across these anorectal indications found that adverse events were uncommon and self-limiting, most often transient faecal incontinence or perianal discomfort, with no episodes of systemic toxicity [35].

Two inconsistencies within this literature deserve to be drawn out rather than mentioned in passing. The first is that the report cited above [33], in which the response did not depend on whether anorectal manometry demonstrated a nonrelaxing internal sphincter, sits uneasily with the physiological rationale for the treatment and with the widespread practice of selecting patients manometrically. If manometry does not predict response, then one of three explanations must hold. Either the relevant mechanism is not confined to sphincter nonrelaxation, or manometry as currently performed lacks the sensitivity to identify the children who will benefit, or part of the improvement seen in uncontrolled series reflects the natural history of functional constipation together with the intensified bowel management, disimpaction, and family engagement that surround the procedure. This question is unresolved, and it matters directly, because it determines whether manometry should remain a gateway to treatment. The second inconsistency is that the only comparative evidence in this field, the meta-analysis of Friedmacher and Puri, favours posterior myectomy over toxin for durable response and reports more nonresponse and more subsequent surgery after toxin [30], a result that the confident tone of the uncontrolled series does not convey.

Evidence and clinical position. The certainty of evidence for intrasphincteric botulinum toxin in functional constipation and internal anal sphincter achalasia is low to moderate. Two small randomised trials support short-term symptomatic and manometric improvement, while the remainder of the evidence consists of retrospective cohorts and case series with variable diagnostic criteria, doses spanning a wide range, differing injection sites and numbers of quadrants, and follow-up that is frequently shorter than the expected duration of effect. The variation between reports is better explained by these methodological differences than by any true divergence in drug effect. In practical terms, botulinum toxin is not a first-line treatment for functional constipation, which remains laxative therapy with behavioural bowel management. It is best positioned as a second-line, reversible intervention in the subgroup with demonstrated or strongly suspected internal anal sphincter dysfunction that has not responded to optimised medical treatment, and as an alternative to myectomy that avoids irreversible division of the sphincter at the cost of a lower rate of durable response.

### 4.2. Persistent Obstructive Symptoms in Hirschsprung Disease

Even after a technically successful pull-through operation for Hirschsprung disease, a proportion of children continue to have obstructive symptoms because the internal anal sphincter remains nonrelaxing. Langer and Birnbaum first reported that intrasphincteric botulinum toxin could relieve this persistent constipation [36], and Minkes and Langer subsequently confirmed the benefit in a prospective study of children with internal anal sphincter hypertonicity after surgery [37]. Koivusalo et al. found the technique useful for anal outlet obstruction in both internal anal sphincter achalasia and Hirschsprung disease [38], and Church et al. showed that ultrasound-guided injection reliably relieved obstructive defecation in these groups [39]. A systematic review and meta-analysis by Roorda et al. concluded that botulinum toxin injection after surgery for Hirschsprung disease reduces obstructive symptoms in the majority of children, while emphasising that the evidence rests largely on observational studies and that the effect is temporary [40].

Consistent with this, a single-institution study found that intrasphincteric toxin reduced the rate of hospitalisation for postoperative obstructive symptoms in children with Hirschsprung disease, which supports its use as a first-line measure when nonrelaxation of the internal sphincter is the cause [41].

Evidence and clinical position. The certainty of evidence here is low. No randomised trial has been performed, and the pooled estimate rests on observational studies with differing definitions of obstructive symptoms and short follow-up, as the authors of the systematic review themselves emphasise. Botulinum toxin is nevertheless reasonably positioned as an early, reversible, diagnostic and therapeutic step in the child with persisting obstructive symptoms after pull-through once mechanical obstruction, transition zone pull-through, and enterocolitis have been excluded, and its temporary effect is an argument for using it as a test of the sphincteric hypothesis before considering myectomy or revision surgery rather than as a durable solution.

### 4.3. Chronic Anal Fissure

Chronic anal fissure in childhood is maintained by spasm and hypertonia of the internal anal sphincter, and the same chemical relaxation used in adults can promote healing without the risk of permanent incontinence that accompanies lateral sphincterotomy. In a paediatric pilot study, Husberg et al. found that injection of botulinum toxin healed chronic fissures in children with only transient and minor adverse effects, although the procedure was performed under light anaesthesia [42]. Keshtgar et al. described a needle-free transcutaneous technique that treated both constipation and anal fissure in children, broadening the appeal of the approach by avoiding a needle in an anxious child [43]. Across the paediatric literature, the treatment is minimally invasive and reversible, but experience remains limited to small series, recurrences occur, and repeat injection is sometimes required, so that toxin is best regarded as a useful step before considering sphincterotomy rather than a definitive cure [6].

Evidence and clinical position. The certainty of evidence in children is very low, resting on small uncontrolled series and on extrapolation from much larger adult literature in which toxin heals fewer fissures than lateral internal sphincterotomy but carries no risk of permanent incontinence. In children, in whom the lifetime consequences of a continence injury are greatest, toxin is appropriately positioned as an intermediate step for fissures that have failed conservative treatment and topical agents and ahead of any sphincter-dividing operation.

### 4.4. Oesophageal Achalasia and Upper Gastrointestinal Uses

Achalasia is rare in children, and although pneumatic dilation, laparoscopic Heller myotomy, and peroral endoscopic myotomy are the definitive treatments, injection of botulinum toxin into the lower oesophageal sphincter offers temporary relief that can be valuable in selected situations. In the largest paediatric series, Hurwitz et al. treated 23 children and found that most responded initially, with a mean duration of effect of about four months and a frequent need for repeat injection [44]. Ip et al. similarly reported symptomatic relief in children, with a sustained response beyond six months in a minority of patients [45]. Because the benefit is short-lived, toxin is generally reserved for children who are poor candidates for more durable procedures or is used as a bridge to definitive treatment, a position supported by adult randomised data showing that laparoscopic Heller myotomy provides more lasting relief than toxin injection [46]. Botulinum toxin has also been used at the other end of the foregut, where injection into the cricopharyngeus can relieve the rare upper oesophageal sphincter dysfunction of cricopharyngeal achalasia in children [47].

The place of the toxin within the wider treatment ladder has become clearer. A two-centre paediatric review found that laparoscopic Heller myotomy gave more durable relief than either balloon dilation or botulinum injection [48], and outcome studies in children have consistently shown that the benefit of injection is short-lived compared with myotomy [49]. With the more recent adoption of peroral endoscopic myotomy, which provides durable relief in children [50], botulinum toxin has settled into a narrower role as a temporising or diagnostic measure rather than a definitive treatment [49].

Beyond the sphincters, botulinum toxin has been injected into the pylorus for gastroparesis, and a paediatric series by Rodriguez et al. found intrapyloric injection to be safe and, in selected children such as older patients and those presenting with vomiting, of symptomatic benefit, although controlled data in children remain lacking [51].

Evidence and clinical position. For oesophageal achalasia the certainty of evidence in children is low, and the direction of that evidence is consistent, since every comparative paediatric report and the adult randomised data agree that myotomy and dilation give more durable relief. Botulinum toxin is therefore positioned as a temporising or bridging intervention, appropriate for the child who is unfit for or awaiting definitive treatment, or where the diagnosis is uncertain and a short-lived response would be informative. For intrapyloric injection in gastroparesis, the certainty is very low, with no controlled paediatric data at all, and the intervention should be regarded as exploratory and offered within a structured follow-up or research setting rather than as established care.

## 5. Urological Applications

The lower urinary tract is a major field of paediatric botulinum toxin therapy, and it contains the one indication, neurogenic detrusor overactivity, for which the toxin has achieved formal regulatory approval in children. Two broad strategies are used. Injection into the detrusor muscle relaxes an overactive bladder and lowers storage pressure, protecting the kidneys and improving continence, whereas injection into the external urethral sphincter reduces outlet resistance when the sphincter fails to relax during voiding [6]. One qualification applies to the phrase protecting the kidneys, which we use here and elsewhere only in a specific sense. Renal protection in this context is inferred from measured reductions in storage pressure and from the well-established relationship between sustained high detrusor pressure and deterioration of the upper urinary tract. No paediatric trial of botulinum toxin has used preservation of renal function, glomerular filtration rate, or new upper tract scarring as a primary endpoint.

### 5.1. Neurogenic Detrusor Overactivity and the Myelomeningocele Bladder

Children with spina bifida and other causes of neurogenic bladder frequently develop a small, high-pressure, overactive detrusor that threatens the upper urinary tract and causes incontinence, and when antimuscarinic drugs fail, the bladder has traditionally been enlarged by augmentation cystoplasty. Injection of botulinum toxin into the detrusor offers a far less invasive alternative. Schulte-Baukloh et al. reported the first paediatric series in 2002, treating children with detrusor hyperreflexia due to myelomeningocele and observing improved bladder capacity and lower storage pressures [52]. A systematic review by Game et al. confirmed that intradetrusor injection produces clinically meaningful gains in capacity, compliance, and continence in children with neurogenic detrusor overactivity, with a very good tolerability profile [53], and a later systematic review focused on spina bifida reached the same conclusion while noting that the supporting studies were mostly small and uncontrolled [54]. A multicentre study extended these observations across several institutions and identified the presence of detrusor overactivity, rather than a poorly compliant fibrotic bladder, as the main predictor of success [55]. The evidence base culminated in a randomised, double-blind, phase three trial by Austin et al., which showed that intradetrusor onabotulinumtoxinA reduced daytime incontinence, lowered maximum detrusor pressure, and increased bladder capacity in children, which supported approval of the toxin by the United States Food and Drug Administration in 2021 for paediatric neurogenic detrusor overactivity at a dose of 200 units not exceeding 6 units per kilogram. The most common adverse events are urinary tract infections and bacteriuria, and because the benefit is temporary, repeat injection is usually required [56].

The paediatric evidence has accumulated steadily since those first reports. Schulte-Baukloh et al. confirmed in a European series that detrusor injection reduced bladder spasticity and raised capacity in children with neurogenic bladder [57], and Riccabona et al. showed the approach to be a safe alternative to more invasive surgery in myelomeningocele [58]. Because the effect is temporary the treatment is repeated, and Altaweel et al. demonstrated that successive injections retain their efficacy without loss of response [59]. A central goal is to lower storage pressure enough to protect the kidneys and to defer or avoid augmentation cystoplasty, and series such as that of Kajbafzadeh et al. reported improvement in both bladder and associated bowel dysfunction after intravesical injection in children with myelomeningocele [60]. Where onabotulinumtoxinA is unsuitable or unavailable, abobotulinumtoxinA has been used with comparable urodynamic benefit that is maintained across repeated injections [61]. A large cohort has since clarified which children respond best, identifying the presence of detrusor overactivity as the strongest predictor of success [62], whereas the small, poorly compliant and fibrotic bladder without overactivity responds poorly, an important limit to patient selection [63].

Further cohorts have addressed patient selection and consolidated the evidence. Khan et al. confirmed benefit in children with a medically refractory neuropathic bladder while noting that not all patients respond [64], Kim et al. identified preoperative urodynamic factors that predict a good response [65], and a systematic review by Wu et al. concluded that intradetrusor injection is effective and safe for refractory neurogenic bladder in children while calling for higher quality controlled studies [66].

Evidence and clinical position. This is the strongest evidence base in paediatric botulinum toxin therapy, and we grade the certainty as moderate. It rests on one adequately powered, randomised, double-blind, placebo-controlled phase three trial, supported by systematic reviews of consistent observational cohorts and by regulatory approval. We grade it moderate rather than high because a single pivotal randomised trial underpins that approval, because its endpoints were daytime incontinence episodes and urodynamic variables rather than renal outcomes, and because data on repeated injection across many years of childhood remain observational. Clinically, botulinum toxin is a second-line treatment, indicated when antimuscarinic or beta three agonist therapy combined with clean intermittent catheterisation has failed or is not tolerated. It serves both as a repeatable alternative to augmentation cystoplasty and, in some children, as a bridge that defers augmentation, and in neither role does it replace continued surveillance of the upper urinary tract.

### 5.2. Idiopathic (Non-Neurogenic) Overactive Bladder

When children have an overactive bladder without any neurological cause and their urgency, frequency, and daytime incontinence persist despite urotherapy and antimuscarinic or beta-three agonist medication, intradetrusor injection can be considered. Hoebeke et al. provided the landmark paediatric report, showing that injection into the detrusor relieved incontinence in a substantial proportion of children with a therapy-resistant overactive detrusor [67]. A large retrospective series later confirmed both effectiveness and safety, while making clear that the response in idiopathic overactive bladder is generally lower and less durable than in the neurogenic setting and that carefully selected patients benefit most [68]. Because the condition is not neurogenic and often improves as the child matures, the toxin is reserved for genuinely refractory cases [6].

Additional paediatric series support this cautious role. Marte et al. found intravesical injection effective for refractory overactive bladder in children who had failed antimuscarinics [69], and a ten-year single-centre experience of both intravesical and intrasphincteric injection confirmed that the toxin is a useful and repeatable option across a range of paediatric bladder and sphincter problems, albeit with effects that wane and require retreatment [70].

Evidence and clinical position. The certainty of evidence in idiopathic overactive bladder is low. No randomised paediatric trial has been conducted; response rates in the reported series are consistently lower and less durable than in the neurogenic setting, and because idiopathic overactive bladder frequently improves with maturation, the uncontrolled design of these studies makes spontaneous resolution difficult to separate from treatment effect. Botulinum toxin therefore belongs at the third line, reserved for children with genuinely refractory symptoms after standardised urotherapy and an adequate trial of pharmacotherapy, and the temporary nature of the response should be made explicit to families before treatment.

### 5.3. Detrusor Sphincter Dyssynergia and Dysfunctional Voiding

A different problem arises when the external urethral sphincter contracts inappropriately during voiding, whether because of a neurological lesion, in which case the pattern is termed detrusor sphincter dyssynergia, or in the absence of any lesion, in which case it is called dysfunctional voiding. The resulting high voiding pressures and incomplete emptying predispose to infection and upper tract damage. Injection of botulinum toxin into the external sphincter relaxes the outlet and can restore more efficient voiding. Steinhardt et al. were the first to apply the technique in a neurologically normal child with refractory dysfunctional voiding, achieving resolution of infections and incontinence [71]. Subsequent paediatric experience, largely in children who have failed biofeedback and behavioural retraining, suggests that sphincter injection can interrupt the cycle of dyssynergic voiding and provide a window during which normal voiding may be relearnt, although the effect is temporary and the evidence remains limited to small series [6].

Several paediatric series bear this out. Radojicic et al. treated children with refractory dysfunctional voiding by transperineal external sphincter and pelvic floor injection combined with biofeedback, and found that the temporary paralysis broke the cycle of dyssynergic voiding and created a window for retraining [72]. Mokhless et al. reported improvement after urethral sphincter injection in children with a nonneurogenic neurogenic bladder [73], Franco et al. described benefit in neurologically normal children with external sphincter dyssynergia [74], and a longer-term study by Vricella et al. confirmed durable efficacy across repeated treatments [75].

Evidence and clinical position. The certainty of evidence is very low, since the entire paediatric literature for this indication consists of small uncontrolled series without blinded outcome assessment, and dysfunctional voiding is precisely the setting in which behavioural intervention, attention, and regression to the mean are most likely to produce apparent improvement. Botulinum toxin is best positioned not as a treatment in its own right but as a temporary adjunct that creates a window of reduced outlet resistance within which biofeedback and voiding retraining can be delivered, and it should follow rather than replace an adequate trial of that retraining.

### 5.4. Posterior Urethral Valves and the Valve Bladder

Boys born with posterior urethral valves often continue to have bladder dysfunction after the obstructing valves have been ablated, a condition known as the valve bladder that may combine detrusor overactivity, poor compliance, and impaired emptying and that places renal function at risk. In principle, the same detrusor or sphincter-directed injections used for other forms of bladder dysfunction can be applied here, and intradetrusor toxin has been used to lower storage pressure in the overactive, poorly compliant valve bladder on the same rationale as in neurogenic detrusor overactivity [53]. Dedicated evidence in this specific group is confined to a single small randomised trial of bladder neck injection, discussed below, as treatment decisions rest largely on extrapolation from the wider experience of paediatric detrusor overactivity, so that botulinum toxin is best regarded as an adjunct within a careful programme of surveillance rather than an established standard for the valve bladder [6].

A small randomised study specific to this group allocated twenty boys after valve ablation to bladder neck injection or to cystourethroscopy alone, ten in each group, and found no difference between them in urinary tract infection, hydronephrosis, vesicoureteral reflux, serum creatinine, cystometric capacity or voiding pressure, the authors concluding that temporarily abolishing bladder neck function does not appear to improve outcome in this group [76].

Evidence and clinical position. The certainty of evidence in the valve bladder is low, resting on one small randomised trial that found no benefit, together with extrapolation from neurogenic detrusor overactivity, and the two conditions differ in that the valve bladder frequently combines overactivity with poor compliance and impaired emptying, so that lowering detrusor pressure may not address the dominant problem and may worsen emptying. Bladder neck injection is not supported by the only randomised evidence available and should not be offered for this purpose, while intradetrusor injection in the valve bladder rests on extrapolation alone and remains an adjunct of unproven benefit within a structured surveillance programme.

## 6. Neurological and Neuromuscular Applications

Neurology is where paediatric botulinum toxin therapy began, and it remains the field in which the toxin is used most often. The overwhelming majority of injections given to children are for the relief of spasticity and other movement disorders of cerebral origin, and the accumulated clinical experience here is larger than in any other indication [6,9].

Before the individual indications are considered, one distinction must be made explicit, because it governs how every study in this section should be read. Reduction in tone and improvement of function are not the same outcome. Within the framework of the International Classification of Functioning, Disability and Health, a fall in the Modified Ashworth or Modified Tardieu score is a change at the level of body structure and function. Walking speed, gross motor performance measured on the Gross Motor Function Measure, independence in daily activities, and participation at school or in play sit at the levels of activity and participation, and a change at the first level does not reliably produce a change at the others. Spasticity is only one contributor to the motor disability of cerebral palsy, which also reflects weakness, impaired selective motor control, poor balance, altered muscle morphology, and the differential growth of muscle, tendon, and bone. Weakening a spastic muscle removes one contributor while potentially unmasking another, since the injection that reduces resistance to passive stretch also reduces the force the muscle can generate. This is precisely why a treatment can be established as effective for spasticity while its effect on function remains uncertain, and it explains the apparent tension between the conclusion of the American Academy of Neurology quoted below and the trial results that follow it. Throughout this section we therefore state, for each body of evidence, whether the outcome reported was a tone-level measure or an activity-level or participation-level measure.

### 6.1. Spasticity in Cerebral Palsy, Lower Limb and Gait

Cerebral palsy is the commonest cause of physical disability in childhood, and focal spasticity of the calf muscles producing an equinus, toe-walking gait is its most frequent motor problem. The pioneering report by Koman et al. in 1993 showed that injection of type A toxin into the overactive muscles reduced tone and improved the dynamic component of the deformity, and this observation launched what has become the largest body of paediatric toxin experience [8]. An evidence-based review by the American Academy of Neurology later classified type A toxin as an established and effective treatment for reducing spasticity in the lower limb, while noting that its effect on function, as distinct from tone, was less certain [9,77]. Practice has been standardised by European consensus recommendations and best practice statements that define weight-based dose ceilings, injection technique, and the integration of toxin with physiotherapy, casting, and orthoses [21,78]. Randomised trials have continued to refine the evidence, including a large randomised placebo-controlled trial of abobotulinumtoxinA in 241 children, in which the Modified Ashworth Scale improved against placebo by 0.49 points at 15 units per kilogram per leg, with a 95 per cent confidence interval of 0.23 to 0.75, and by 0.38 points at 10 units per kilogram per leg, with an interval of 0.13 to 0.64, both measured at four weeks, alongside a corresponding improvement in the Physician’s Global Assessment. It should be noted that both of these endpoints measure tone and clinician impression rather than validated function [79]. In practice the toxin does not cure the underlying disorder but buys time, delaying fixed contracture and orthopaedic surgery during the years of most rapid growth, and it is used repeatedly within a multidisciplinary programme rather than as a single intervention [21,22].

The strength of the evidence in the lower limb comes from a series of randomised controlled trials. An early preliminary double-blind, placebo-controlled trial by Koman et al. in only twelve ambulatory children, six given toxin and six placebo at a dose of one unit per kilogram per leg, reported improvement on the authors’ own Physician Rating Scale in five of six treated children against two of six given placebo, and those authors themselves concluded that the finding merited further prospective study. That trial should be read with three further facts in view. The placebo group also improved, by an average of 2.3 points on the fourteen-point scale, against 3.1 points in the treated group. Isokinetic dynamometry showed no consistent difference between the groups. No statistical testing of the comparison was reported [80], and a larger placebo-controlled trial by the same group confirmed that type A toxin reduced equinus and improved gait scores [81]. A double-blind, placebo-controlled dose-ranging study in 125 children compared 10, 20 and 30 units per kilogram of abobotulinumtoxinA injected into the gastrocnemius. All three doses significantly reduced the dynamic component of gastrocnemius shortening compared with placebo at four weeks, and the effect was most pronounced at 20 units per kilogram, where the mean change in the dynamic component of 1.84 per cent corresponds, on that trial’s own conversion of 10.6 degrees per one per cent, to about 19 degrees of dorsiflexion available during walking. That figure is a model-derived equivalent and not a measured joint angle. Measured passive dorsiflexion with the knee extended did not change significantly in that group, and maximum passive gastrocnemius length showed no significant between-group difference at any dose. The effect on the dynamic component was still present at sixteen weeks. That trial also used the Gross Motor Function Measure as its only standardised functional instrument, and it showed no statistically significant difference from placebo at either four or sixteen weeks, on the overall score or on the goal total score, while parent and investigator ratings of functional benefit were markedly more favourable in every active group at every timepoint [82], and instrumented three-dimensional gait analysis confirmed improvement in ankle kinematics after injection, with dorsiflexion at ten per cent of the gait cycle of 5.4 degrees against minus 3.4 in controls, peak dorsiflexion in stance of 12.5 against minus 3.6, and peak dorsiflexion in swing of 6.2 against minus 3.8. Those gains were confined to ankle joint angle. Ankle alignment at foot contact did not differ significantly, and there were no significant differences in step length, stride length, cadence, or walking velocity, so an improvement in joint kinematics did not translate into measured walking performance [83]. A further randomised, double-blind, placebo-controlled trial in 40 children found significant improvement in initial foot contact on video gait analysis, which was its primary endpoint, at six and twelve weeks, with clinical improvement in 48 per cent of treated children against 17 per cent of those given placebo, and a significant gain in the walking dimension of the Gross Motor Function Measure. In the same trial the physiological cost index and passive ankle dorsiflexion did not change significantly. That trial recruited 40 children against the 56 its own calculation required, giving roughly 70 per cent power, and the groups were imbalanced at entry in the direction of the reported effect, the placebo group having a median Gross Motor Function Measure standing score of 71.8 against 85.9 and a walking and running score of 54.2 against 69.4, with three hemiplegic children against nine, so the more able children were in the treated arm [84], and when compared directly with serial casting in a randomised prospective trial of 20 children the toxin was of similar efficacy, with the tone reduction it produced allowing a more prolonged improvement in passive dorsiflexion than casting achieved [85].

Functional rather than purely tone-related outcomes have also been examined, and the most instructive trial in that respect has to be read carefully. In a randomised crossover trial, Reddihough et al. found no difference in the Gross Motor Function Measure between the injected phase and the control phase at either three or six months, across any of its ten dimensions, and at three months the mean difference favoured the control phase in half of them. That null result is uninformative rather than reassuring, because the authors report a post hoc power calculation showing that with nineteen children the power to detect a seven per cent change in the total score was only 0.25. Three further features limit the comparison. There was no washout period at the six-month crossover, although those authors state that the toxin weakens muscle for three to six months, so the control phase of the first group began immediately after its treated phase. Physiotherapy was not held constant between phases, averaging 27.8 h during the toxin phase against 20.9 h during the control phase. Twelve of sixty-one children did not complete, seven of them withdrawn because they required surgery during the study, which is plausibly related to an inadequate response. The one positive functional finding, a significant increase in the fine motor rating of the Vulpe Assessment Battery, was obtained in eighteen children at a single timepoint on an upper limb measure within a trial of lower limb injection, and it emerged from a large family of comparisons without adjustment for multiplicity, so it should not be presented as a functional benefit of lower limb treatment. Parents nonetheless rated the benefit of treatment highly at both three and six months, most of them reporting maximum benefit by about six weeks, which is before the first formal assessment [86]. The divergence between a favourable parental impression and a neutral blinded measure recurs throughout the paediatric literature and is taken up again below. Because spasticity in cerebral palsy is seldom confined to one muscle, multilevel injection combined with structured rehabilitation has become standard, an approach that a randomised trial by Scholtes et al. showed to improve mobility, with estimated between-group gains in the GMFM-66 of 2.07 points at twelve weeks and 3.48 points at twenty-four weeks, both above the 1.6 points that trial itself defined as clinically meaningful, although the lower confidence limit at twelve weeks of 0.31 falls below that threshold. That trial does not isolate the contribution of the injection, and its authors do not claim that it does. The treated arm received a standardised programme of three to five physiotherapy sessions a week of 45 to 60 min over twelve weeks, with serial casting where indicated and prescribed orthoses, whereas the control arm continued usual care of one to two sessions of 30 to 60 min a week, so the treated children received roughly three to four times the physiotherapy exposure during the comparison window. Those authors also state that observers could not be blinded, because the differing number of assessments made allocation obvious, so the Gross Motor Function Measure was scored unblinded and the problem score was reported by unblinded parents. The one instrumented measure, the energy cost of walking by gas analysis, was null at every timepoint, although that comparison rests on a subgroup of twenty-four children at a single centre [87].

The functional claims in the preceding two paragraphs require qualification, and we set them beside the tone-level results deliberately rather than allowing them to read as repeated demonstrations of functional benefit. The trials cited above used heterogeneous instruments, including the Modified Ashworth Scale and Modified Tardieu Scale for tone, the Physician Rating Scale and Observational Gait Scale for gait pattern, instrumented three-dimensional gait analysis for ankle kinematics, and the Gross Motor Function Measure for gross motor performance, and the magnitude of effect was consistently larger for tone-level than for activity-level outcomes. Where a positive functional result was reported, it was usually a secondary endpoint within a trial powered for a tone-level primary endpoint, the effect size was modest, and follow-up rarely extended beyond one or two injection cycles. Table 2 sets out the design, outcome instruments, effect estimates, and follow-up duration of the studies that reported activity-level outcomes alongside tone, since these are the studies that bear directly on the distinction being drawn here.

Studies designed with function as the primary endpoint and with longer follow-up have been distinctly less encouraging. In a randomised study of fifteen toddlers with spastic cerebral palsy, plantarflexor tone fell significantly from baseline within the treated group at three and a half years, but there was no significant between-group difference in the change in plantarflexor tone at any timepoint, and the only between-group difference to reach significance was in the change in popliteal angle. That trial was powered to detect a five-degree difference in ankle dorsal extension, and that outcome was null at every timepoint. No between-group differences were found in three-dimensional gait analysis, in the Gross Motor Function Measure, or in the Paediatric Evaluation of Disability Inventory, and the authors concluded that the effect on gait development remained inconclusive. The randomised comparison in that trial also does not survive the first year, because five of the nine control children began botulinum toxin treatment during follow-up, so that by the three and a half-year endpoint the control group had itself been substantially treated [88]. In a prospective cohort of 124 ambulant children aged two to six years in Gross Motor Function Classification System levels I to III followed over three years, repeated injections produced no significant difference in GMFM-66 scores between treated and untreated children, and no preferential effect on passive range of motion was seen. The small differences in passive ankle dorsiflexion and in hamstring range that favoured the comparison group, of 4.17 and 4.96 degrees, are of similar magnitude to the baseline imbalances in the same direction, which reached 4.86 and 7.79 degrees, so they cannot be attributed to the intervention. The gross motor comparison is the more secure of that study’s findings, since the two groups did not differ significantly on the GMFM-66 at entry, and both pre-planned sensitivity analyses reproduced the result. Two features nevertheless limit how far the study can be used as negative evidence. Allocation was by clinical decision and the treated children were the more impaired at entry, with a median passive ankle dorsiflexion of 6 degrees against 10 and a median daily step count of 4204 against 7294, so residual confounding would tend to make the toxin appear worse rather than better and a genuine functional benefit cannot be excluded. Assessments were also made at least three months after the most recent injection, when the effect has largely resolved, so the study tests durable or cumulative change rather than acute response [89]. In the upper limb, a systematic review and meta-analysis of fourteen randomised trials in 621 children, appraised with the revised Cochrane risk-of-bias tool and with GRADE, confirmed a reduction in muscle tone, with a pooled mean difference in wrist Ashworth score of −0.85 and a 95 per cent confidence interval of −1.42 to −0.27, while finding no significant functional gain, the Melbourne Assessment mean difference being 3.13 with a confidence interval of −3.13 to 9.30 and the Assisting Hand Assessment mean difference 3.84 with a confidence interval of −1.86 to 9.54, and concluding that functional benefit is not clearly established and that the discrepancy between subjective and objective results requires further investigation [90]. These findings support the position of the American Academy of Neurology quoted above rather than contradicting it. The reasonable expectation from botulinum toxin in cerebral palsy is a reliable but temporary reduction in focal tone, together with functional gain that is possible, individually variable, and heavily dependent on the rehabilitation delivered alongside the injection.

**Table 2:** Studies of botulinum toxin in cerebral palsy that reported activity-level outcomes alongside tone, with design, outcome instruments, effect estimates, and follow-up.

Study	Design and Sample	Primary Outcome and ICF Level	Activity-Level Measures Used	Result	Follow-Up
Tedroff et al. [88]	Randomised controlled study, 15 children with spastic cerebral palsy, mean age 16 months	Plantarflexor muscle tone, body structure and function level. Assessed unblinded, whereas the three-dimensional gait analysis was blinded	Three-dimensional gait analysis, GMFM-66, PEDI	Plantarflexor tone fell from baseline within the treated group at 3.5 years, but there was no significant between-group difference in tone change at any timepoint. The only significant between-group difference was in popliteal angle change. The outcome the trial was powered for, a 5-degree difference in ankle dorsal extension, was null at every timepoint. No between-group difference in three-dimensional gait analysis, GMFM-66 or PEDI. Five of the nine control children commenced botulinum toxin during follow-up, so the control group was substantially treated by the endpoint	3.5 years, gait analysis at age 5
Fehlings et al. [89]	Prospective observational cohort with a non-randomised comparison group, 124 children aged 2 to 6 years, GMFCS levels I to III, 65 treated and 59 comparison	GMFM-66, activity level	Passive ankle dorsiflexion with knee extended, additional activity and participation measures	No significant group difference in GMFM-66, effect 0.92, standard error 0.81, 95 per cent CI −0.66 to 2.50, *p* = 0.256, a result reproduced by both pre-planned sensitivity analyses and not confounded by baseline imbalance on that measure. No preferential effect on passive range of motion. The differences favouring the comparison group, 4.17 degrees for ankle dorsiflexion and 4.96 degrees for hamstring range, are of similar magnitude to baseline imbalances in the same direction of up to 4.86 and 7.79 degrees, so they cannot be attributed to the intervention. Daily step counts favoured the treated group without reaching significance. Allocation was by clinical decision and the treated children were more impaired at entry, so residual confounding runs against the treated group	3 years
Upper limb systematic review and meta-analysis [90]	14 randomised controlled trials, 621 children, 4862 records screened, appraised with the revised Cochrane risk-of-bias tool and GRADE	Muscle tone on the Ashworth scale, body structure and function level	Melbourne Assessment of Unilateral Upper Limb Function, Assisting Hand Assessment, Goal Attainment Scaling, Canadian Occupational Performance Measure	Wrist Ashworth mean difference −0.85, 95 per cent CI −1.42 to −0.27. No significant functional gain, Melbourne Assessment mean difference 3.13, 95 per cent CI −3.13 to 9.30, and Assisting Hand Assessment mean difference 3.84, 95 per cent CI −1.86 to 9.54. The authors concluded functional benefit is not clearly established and noted a divergence between subjective and objective results	Varied by trial
Reddihough et al. [86]	Randomised crossover trial, 49 of 61 recruited children, aged 22 to 80 months, injection plus physiotherapy versus physiotherapy alone	Gross Motor Function Measure, activity level	Vulpe Assessment Battery, joint range of movement, parental questionnaire	Sustained gross motor gains in both groups. The only additional benefit attributable to injection was the fine motor rating on the Vulpe Assessment Battery. Parents rated benefit highly despite the neutral objective result	6 months per arm

ICF, International Classification of Functioning, Disability and Health. GMFM-66, 66-item Gross Motor Function Measure. PEDI, Paediatric Evaluation of Disability Inventory. GMFCS, Gross Motor Function Classification System. CI, confidence interval.

Not every application has succeeded, and an honest account includes the negative trials. Cosgrove et al. established early that the toxin could be used to manage the lower limb in cerebral palsy [91], yet a randomised controlled trial by Graham et al. found that toxin combined with bracing did not prevent progressive hip displacement in children with hips at risk, a reminder that chemodenervation does not alter the bony consequences of persistent muscle imbalance [92].

Evidence and clinical position. We grade the certainty of evidence as high for the reduction in focal lower limb spasticity and as low for improvement in gross motor function, gait quality, or participation. It is worth being explicit about what the high rating for tone rests on. It rests on two adequately powered placebo-controlled trials, of 241 and 126 children randomised, respectively, both of which we judge to be at low risk of bias on every domain of the revised Cochrane tool. They are the only two trials in this indication that we so judge. Each pre-specified its primary outcome and the timepoint at which that outcome would be assessed, and each met or exceeded its own recruitment target. Each reported a significant effect against placebo on its primary physiological measure of the spastic muscle, although the two measures are not the same thing. The larger trial measured muscle tone directly with the Modified Ashworth Scale, whereas the other measured the dynamic component of gastrocnemius shortening by calibrated electrogoniometry, which is a dynamic muscle-length measure derived from the difference between the range available at rest and the range used while walking. Two further caveats attach to the second of those trials. Its dose response was not monotonic, since 20 units per kilogram outperformed 30, so it supports the existence of an effect rather than a dose-effect relationship, and it analysed legs as separate observations although the two legs of one child are not independent, a limitation its authors acknowledge and check against per-patient summaries with consistent results. The remaining randomised evidence in this indication comes from trials of between twelve and forty children, several of which we judge to be at some concern or at high risk of bias, and it should not be read as adding independent weight to that estimate. The low rating for function reflects a related asymmetry. The one trial we judge to be at low risk of bias used no standardised functional instrument, its endpoints being muscle tone and a clinician global impression, whereas the two trials that did measure walking performance with instruments returned null results for step length, stride length, cadence, walking velocity, the physiological cost index and passive ankle dorsiflexion. Taken across the indication as a whole, the appraised evidence supports the low rating for function more firmly than any single trial does. Of the eleven primary studies, the two we judge to be at low risk of bias both used physiological rather than functional primary endpoints, and in one of them the sole standardised functional instrument was null against placebo. With one exception, the functional gains reported in this group were either scored unblinded, as in the multilevel rehabilitation trial and in the crossover trial, or confounded with a substantially larger concurrent rehabilitation dose, as in the former. One trial does report a gain on a standardised functional instrument against placebo, in the walking dimension of the Gross Motor Function Measure, but it did so as a secondary outcome in a trial whose groups were imbalanced at entry in the direction of the reported effect and whose recruitment fell short of its own power calculation. Botulinum toxin is a first-line focal treatment for dynamic, focal lower limb spasticity in the ambulant child, positioned as an adjunct within a programme of physiotherapy, casting, and orthotic management, and as a means of deferring orthopaedic surgery through the years of rapid growth rather than of preventing it. It has no established role in correcting fixed contracture, and the randomised evidence for the prevention of hip displacement is negative.

### 6.2. Spasticity in Cerebral Palsy, Upper Limb

In the haemiplegic child the spastic upper limb, typically a flexed wrist and pronated forearm with the thumb held in the palm, interferes with reaching and grasping. A Cochrane review concluded that type A toxin used together with occupational therapy reduces upper limb tone and assists the achievement of individualised goals, although the effect on overall function is modest [93]. An international consensus statement has provided a framework for assessment, muscle selection, and aftercare specific to the upper limb [94], and a large randomised repeat treatment study of abobotulinumtoxinA confirmed sustained reduction in spasticity and goal attainment across successive injection cycles combined with a home exercise programme [95]. As in the lower limb, the toxin is an adjunct that opens a therapeutic window for active training rather than a treatment used on its own [94].

Because goal attainment recurs throughout the upper limb literature, its nature should be made explicit for readers outside rehabilitation medicine. The instrument referred to is Goal Attainment Scaling, in which child, family, and therapist agree in advance on a small number of individualised goals and define a five-point scale of possible outcomes for each, from much less than expected to much more than expected, from which a standardised T score is derived. Goal Attainment Scaling is responsive and clinically meaningful precisely because it measures what matters to the particular family, but for the same reason the goals differ from child to child and from study to study. A Goal Attainment Scaling T score is therefore not comparable across children, centres, or trials in the way that a Gross Motor Function Measure score or a walking speed is, and it cannot be pooled straightforwardly in meta-analysis. Goals are also usually rated by people aware of the treatment given, and the divergence between favourable subjective ratings and neutral objective measures in the upper limb literature has been noted explicitly in meta-analysis [90]. High rates of goal attainment should accordingly be read as evidence that individually chosen aims were met, and not as evidence of a generalisable functional gain.

Several randomised trials underpin the upper limb evidence. Fehlings et al. found that injection improved upper extremity function in children with haemiplegic cerebral palsy [96], a randomised trial by Speth et al. showed added benefit when the toxin was combined with intensive occupational therapy [97], and a further randomised controlled trial by Wallen et al. confirmed functional gains when injection was paired with structured occupational therapy [98].

More recent trials have extended the evidence to newer products, and a randomised phase 3 trial of incobotulinumtoxinA in 350 children and adolescents aged two to seventeen years, spanning all Gross Motor Function Classification System levels, found that Ashworth Scale scores improved in every dose group, with a significantly greater reduction at 8 units per kilogram than at 2 units per kilogram, and that global impression of change improved on investigator, child and parent ratings, with benefit sustained across three further open-label cycles and no increase in adverse events with either higher dose or repeated treatment. Two features of that trial should be kept in view. It compared three active doses rather than including a placebo arm, so the lowest dose served as a comparator rather than a true control, and its endpoints were muscle tone and global impression rather than validated measures of function [99].

The double-blind randomised trial of Corry et al., in fourteen children with seven per arm, was among the first to examine injection in the haemiplegic upper limb, and its results are considerably more mixed than they are usually reported to be. At two weeks the significant between-group differences were wrist resonant frequency, active elbow extension, active thumb extension, elbow tone and wrist tone, whereas thumb abduction, wrist extension, metacarpophalangeal extension, thumb tone, grasp and release, and the coin transfer test of fine motor function were all not significant. By twelve weeks only wrist resonant frequency, wrist tone and grasp and release remained significant, the gains in elbow extension, thumb extension and elbow tone having been lost. The tone and function results rest on one-tailed Fisher exact tests, which place the whole of the alpha in the direction of benefit, applied across twenty-two comparisons in seven children per arm with no adjustment for multiplicity. The coin transfer test was not significant at either timepoint and its range included deterioration, which those authors report as temporary worsening in some children. Their own summary is that the median changes were small although the range was large, and that the most notable subjective change was the cosmetic benefit of reduced involuntary elbow flexion [100], and subsequent randomised trials have refined how the toxin is combined with therapy and how it is dosed. In a population-based single-blind randomised trial of 43 children, Russo et al. found that injection added to occupational therapy significantly reduced tone at the elbow and wrist on the Modified Ashworth and Tardieu scales at both three and six months, and produced higher Goal Attainment Scaling scores and higher global self-worth at three months. Neither of the latter two advantages persisted at six months, and there was no significant difference at any time point in the Assessment of Motor and Process Skills, the Paediatric Evaluation of Disability Inventory, or the Paediatric Quality of Life Inventory. Self-rated athletic competence was significantly lower in the treated group at three months. The authors described their findings as providing modest support for the intervention [101]. Where the blinding fell in that trial deserves note. The tone assessments on the Modified Ashworth and Tardieu scales were carried out unblinded by the same rehabilitation specialist who had administered the injections, because only one specialist trained in the technique was available, whereas the activity-level, disability and quality-of-life measures were administered by an assessor blind to allocation. The outcomes that improved were therefore those measured without blinding, and the outcomes measured blind showed no difference, a pattern that should temper how the positive tone results are read, a randomised trial by Olesch et al. in 22 young children given three cycles of injection alongside occupational therapy found progressively reduced spasticity in the forearm pronators and wrist flexors and higher parent-rated performance on the Canadian Occupational Performance Measure and on Goal Attainment Scaling at twelve months, but no difference in satisfaction on the Canadian Occupational Performance Measure, in quality of movement on the Quality of Upper Extremity Skills Test, or in fine motor skills on the Peabody Developmental Motor Scales [102]. Read together, these two trials indicate something more specific than a general caution. In both of them the outcomes that showed benefit were those assessed without blinding or reported by parents, while the outcomes administered by a blinded observer showed no difference. In the trial of Russo et al. the tone assessments were unblinded and positive whereas every blinded instrument was null, and in the trial of Olesch et al. the two primary outcomes were measures of parental perception elicited by a therapist who was not blinded, and were positive, whereas the observer-administered measures of movement quality and of fine motor skill were not. This does not establish that the reported benefits are artefacts of assessment, but it does mean that the apparent strength of the upper limb evidence rests substantially on unblinded assessment, which is one of the reasons we grade functional benefit in this indication as being of low certainty. The same pattern is not confined to the upper limb. In the toddler trial discussed in the preceding subsection, the findings that reached significance came from the unblinded assessments of tone and of joint range, whereas the one assessment those authors describe as blinded, the three-dimensional gait analysis, was null [88]. The crossover trial discussed in that subsection shows the same divergence, with a parent questionnaire significantly positive at three and at six months against a videotaped and blindly rated Gross Motor Function Measure that was null [86]. Unblinded assessment is not, however, a sufficient explanation, and we state that because the evidence does not support the simpler claim. In the dose-ranging trial discussed in the preceding subsection, blinding was maintained until after the final assessment and after the statistical analysis plan had been completed, and the divergence appeared nonetheless. Parent and investigator ratings of functional benefit were markedly more favourable in every active group at every timepoint while the Gross Motor Function Measure showed no significant difference from placebo [82]. The same holds in the haemiplegic upper limb trial described above, where parent and patient opinion favoured the toxin markedly at two weeks while blinding remained fully intact, and where the fine motor measure was null at both timepoints [100]. The defensible conclusion is therefore narrower than blinding alone. The apparent functional benefit of botulinum toxin in cerebral palsy rests substantially on subjective and clinician-impression outcomes, to which unblinded assessment is one contributor and the choice of instrument is another. A global impression scale asks a different question from a standardised functional measure, and in this literature it answers that question more favourably, and a double-blind randomised dose comparison trial by Kawamura et al. in 39 children found no difference between a low dose and a dose twice as high, either in the Quality of Upper Extremity Skills Test, in the self-care domain of the Paediatric Evaluation of Disability Inventory, or in grip strength, at one and three months after injection. That result does not identify an optimal dose so much as indicate that the higher dose conferred no additional functional benefit, which argues for using the lowest effective dose in the growing arm [103].

Evidence and clinical position. We grade the certainty as moderate for reduction in upper limb tone and for attainment of individualised goals, and as low for improvement in measured upper limb function on standardised instruments such as the Melbourne Assessment of Unilateral Upper Limb Function or the Assisting Hand Assessment. In the spastic upper limb, botulinum toxin is an adjunct rather than a treatment in its own right, and it should be given only alongside a defined programme of occupational therapy or active training, since every trial reporting functional benefit paired injection with therapy and none demonstrated it for injection alone.

### 6.3. Dystonia

Dystonia, the sustained or intermittent muscle contractions that produce twisting postures and repetitive movements, is more difficult to treat than spasticity and is common in the dyskinetic form of cerebral palsy as well as in inherited and acquired disorders of childhood. Botulinum toxin is a first-line treatment for focal dystonia in adults, but in children the evidence is far weaker and rests largely on open-label series and case reports rather than controlled trials [6]. The most recent systematic review of dystonia in cerebral palsy, prepared as a care pathway for the American Academy for Cerebral Palsy and Developmental Medicine and graded against American Academy of Neurology criteria, extracted only three botulinum toxin studies and rated the evidence for dystonia reduction as level U, meaning the data are inadequate to support or refute an effect, against level C, possibly effective, for intrathecal baclofen and deep brain stimulation. Its authors concluded that current evidence does not support botulinum toxin for reducing dystonia and that clinical use rests on expert opinion, so that treatment is individualised and directed at the most disabling focal component, for example, a cervical or limb dystonia that interferes with care or function. Because dystonia is generated centrally and usually involves many muscles, focal chemodenervation can address only part of the problem, and realistic goals such as easing pain, improving positioning, or facilitating hygiene are more appropriate than an expectation of a cure [104,105].

A dedicated systematic review of botulinum toxin in paediatric dystonia found only uncontrolled studies and case reports, yet these consistently described gains in motor function, reductions in pain, and improved quality of life with few adverse effects, which supports a targeted role for the toxin within a broader plan [106]. Practical guidance for childhood dystonia accordingly positions the toxin as one option alongside oral agents and deep brain stimulation, reserved for the focal features that most impair function [107].

Evidence and clinical position. We grade the certainty as very low, since the paediatric evidence consists entirely of open-label series and case reports without control groups or blinded assessment, and this is consistent with the level U rating returned by the most recent systematic review. Botulinum toxin in childhood dystonia is a targeted, symptom-directed adjunct reserved for the most disabling focal component of a generalised disorder. It is not a treatment for generalised dystonia, for which oral pharmacotherapy and deep brain stimulation carry the greater evidence, and realistic aims are relief of pain, easier positioning, and easier delivery of care rather than improved motor performance.

### 6.4. Considerations in Disorders of Neuromuscular Transmission

Because botulinum toxin acts precisely at the neuromuscular junction, disorders that already impair neuromuscular transmission demand particular caution. In myasthenia gravis, the Lambert-Eaton myasthenic syndrome, and the congenital myasthenic syndromes, the safety margin is reduced and injection may provoke or unmask generalised weakness, so these conditions are regarded as relative or absolute contraindications and the product labelling warns against use in this setting [5,25]. Similar caution applies when a child is receiving aminoglycoside antibiotics or other agents that interfere with neuromuscular transmission, since these can potentiate the effect of the toxin [25]. Motor neurone and peripheral nerve disorders are uncommon in children but warrant the same care. The practical message is that a careful neuromuscular history forms part of the assessment before any injection, and that the presence of a transmission disorder shifts the balance of risk and benefit markedly [6,25].

## 7. Head and Neck Applications

The head and neck contain several distinct paediatric targets for botulinum toxin, from the salivary glands to the extraocular and cervical muscles, and this region includes chronic sialorrhoea, which has become the second indication after neurogenic detrusor overactivity to secure a formal paediatric approval [6].

### 7.1. Sialorrhoea (Drooling)

Excessive drooling is common in children with cerebral palsy and other neurodisabilities, and beyond its social burden, it predisposes to skin breakdown, aspiration, and repeated respiratory infection. Injection of botulinum toxin into the parotid and submandibular glands reduces the volume of saliva by blocking the cholinergic drive to the secretory cells. A randomised trial by Reid et al. showed that salivary gland injection significantly reduced drooling in children with neurological disorders compared with control [108]. The evidence matured with the SIPEXI trial, a randomised, double-blind, placebo-controlled study by Berweck et al. that provided class one evidence for the efficacy and safety of incobotulinumtoxinA in children aged six to seventeen years and led to incobotulinumtoxinA becoming the first botulinum toxin formally approved for chronic sialorrhoea [109]. A systematic review and meta-analysis has confirmed a consistent reduction in drooling across studies in children with cerebral palsy, with a favourable safety profile, the main risks being a transient thickening of saliva and, uncommonly, difficulty swallowing. That review pooled twenty-one trials in eight hundred and twenty-seven participants, of whom five hundred and seventy-three had cerebral palsy, but only four trials carried data suitable for meta-analysis, and the pooled reductions in the drooling quotient and in the drooling frequency and severity scale reached *p*-values of 0.002 and 0.004, respectively. Because sixteen of the twenty-one trials were observational and the quantitative synthesis rests on four of them, the review supports the direction of effect more securely than its magnitude. Ultrasound guidance is used to place the injections accurately within the glands [110].

Controlled data reinforce these observations. In a controlled clinical trial, Jongerius et al. confirmed a significant reduction in drooling after salivary gland injection and found the toxin better tolerated than transdermal scopolamine [111]. Injection of both the parotid and submandibular glands, rather than the submandibular alone, gives a fuller effect, as shown by Banerjee et al. in children with cerebral palsy [112], and a randomised comparison against submandibular duct ligation in fifty-seven children and adolescents, fifty-three of whom entered the intention-to-treat analysis, found that surgery outperformed the toxin at thirty-two weeks, with response rates of 63.0 per cent against 26.9 per cent for a reduction of at least half in the drooling quotient or visual analogue score [113]. That trial establishes the toxin as the less invasive option rather than the more effective one, and its partly single-blinded design, unavoidable when an injection is compared with an operation, is the main threat to its internal validity.

Other controlled data reinforce the effect. A double-blind placebo-controlled trial by Alrefai et al. in twenty-four children aged twenty-one months to seven years found that intraparotid injection reduced the frequency of drooling, its severity, and the total drooling score after the first injection, with *p*-values of 0.034, 0.026 and 0.027, respectively, although the planned analysis of a second injection cycle was abandoned because of dropout in the placebo arm, which limits what the trial can say about repeated treatment [114], and a large series by Scheffer et al. in 131 children documented both the efficacy and the limited duration of the effect, with a median benefit of several months [115].

Evidence and clinical position. We grade the certainty as high. Four randomised paediatric trials, two of them placebo-controlled and one a randomised comparison against submandibular duct surgery, together with a systematic review and meta-analysis, give concordant results, and the SIPEXI trial supported the first formal regulatory approval of a botulinum toxin preparation for chronic sialorrhoea in children aged six to seventeen years. This is therefore among the small number of paediatric indications in this review resting on replicated randomised evidence. The principal limitations are the finite duration of effect, with a median benefit of several months requiring repeated treatment, and a small but real risk of thickened saliva and of dysphagia, which is the reason that ultrasound guidance and conservative dosing of the submandibular glands are advised. Botulinum toxin is the principal pharmacological option once positioning, oral motor therapy and anticholinergic medication have failed or are not tolerated, and it is a less invasive alternative to salivary duct surgery, which produces a larger and more durable effect at the cost of an operation.

### 7.2. Congenital Muscular Torticollis

Congenital muscular torticollis, the commonest cause of a persistently tilted and rotated head in infancy, results from shortening and fibrosis of the sternocleidomastoid muscle, and most cases resolve with physiotherapy and stretching. For the minority that fail conservative care, injection of botulinum toxin into the tight sternocleidomastoid, sometimes together with the upper trapezius, can weaken the muscle enough to allow effective stretching and restore range of motion, offering an intermediate step before surgical release. Joyce and de Chalain reported improved range and posture in infants with recalcitrant torticollis after injection [116], and a large retrospective series by Sinn et al. found successful outcomes in the majority of refractory cases, with only a small proportion ultimately requiring surgery. Because injection close to the swallowing and neck muscles carries a risk of transient dysphagia or neck weakness, careful dosing and accurate localisation are important in this young age group [117].

The paediatric series are consistent. Oleszek et al. reported improved cervical rotation or head tilt in about three quarters of children with recalcitrant torticollis after injection of the sternocleidomastoid or upper trapezius [118], and a combined protocol pairing the toxin with a guided physiotherapy programme reduced the proportion of children who went on to require surgery [119].

Evidence and clinical position. We grade the certainty as very low. The paediatric literature consists of uncontrolled retrospective series and small prospective cohorts with unblinded outcome assessment, and because congenital muscular torticollis resolves with physiotherapy in the great majority of infants, uncontrolled response rates will overestimate the effect attributable to injection rather than to continued stretching and to natural history. Botulinum toxin is positioned as an intermediate step reserved for the minority of infants whose torticollis has not responded to an adequate programme of physiotherapy, its purpose being to weaken the shortened sternocleidomastoid sufficiently to make stretching effective and so to avoid or defer surgical release. Dosing and localisation require particular care in this age group because of the proximity of the swallowing and neck muscles.

### 7.3. Strabismus

Strabismus is where the therapeutic story of botulinum toxin began, and it remains a genuine alternative to incisional surgery in selected childhood squints. Injection into an extraocular muscle weakens it for a few months, during which the eyes may realign, and if binocular vision is achieved, the correction can become lasting [3,4]. A Cochrane review found only low-certainty evidence overall but concluded that the toxin may be useful in particular situations, especially small and moderate angle esotropia [120]. In infantile esotropia, comparative paediatric studies have reported that botulinum toxin can achieve motor alignment comparable in some respects to surgery, although many children need repeat injection or eventual operation [121]. The toxin has proved particularly valuable in acute-onset comitant esotropia, where early injection can restore alignment and protect binocular vision, and here it has compared favourably with surgery in children [122]. Transient ptosis and a vertical deviation from spread to neighbouring muscles are the characteristic side effects and are self-limiting [120].

Comparative paediatric studies have helped to define the place of the toxin. In randomised trials, Tejedor et al. found that, in children needing further treatment after previous surgery for acquired or infantile esotropia, injection achieved motor outcomes comparable to a second operation while avoiding it [123,124]. In acute-onset comitant esotropia, a single-centre retrospective comparison in forty-nine children found injection noninferior to incisional surgery at six months, with a markedly shorter general anaesthetic and a shorter recovery time, which is a meaningful advantage in young children [122], and a subsequent multicentre comparison reported that outcomes remained comparable at three years [125].

The phrase, comparable in some respects, requires specification. What these comparative paediatric studies demonstrate is comparability of motor alignment, meaning reduction in the horizontal deviation measured in prism dioptres and the proportion of children reaching a defined alignment threshold at a stated follow-up point. Sensory outcomes are reported in some but not all of these studies. The two randomised comparisons with reoperation, in 47 and 55 children, respectively, assessed detectable fusion and stereopsis and found no significant difference between injection and further surgery, and the retrospective comparison in acute-onset comitant esotropia defined success as alignment within ten prism dioptres together with evidence of binocular single vision, and reported no significant difference in median stereoacuity between injection and surgery at six or at eighteen months [122]. These sensory data are reassuring, but they come from small samples and are reported inconsistently across the wider paediatric literature, so comparability of alignment should not be read as established equivalence of binocular function. Comparability of motor alignment must also be read together with a higher rate of reintervention after toxin than after incisional surgery, since a proportion of children require repeat injection or eventual operation. The one setting in which a sensory outcome is the explicit aim is acute acquired comitant esotropia, where early injection is directed at protecting binocularity before suppression becomes established, and where sensory results have been reported directly.

Evidence and clinical position. We grade the certainty as low overall, in keeping with the Cochrane assessment, and as moderate in acute acquired comitant esotropia. Botulinum toxin is a reasonable first-line alternative to surgery in small and moderate angle esotropia and in acute acquired comitant esotropia, and an adjunct or repeat option in residual or recurrent deviation after previous surgery, but it is not a substitute for surgery in large angle or restrictive strabismus.

### 7.4. Chronic Migraine and Other Uses

In adults, onabotulinumtoxinA injected according to a fixed protocol is an established preventive treatment for chronic migraine, and its use has been explored in adolescents who carry a similar burden of disability. The paediatric evidence remains limited and mixed, with two randomised trials that reached different conclusions, and a systematic review and a meta-analysis of the combined literature, in which those trials are summarised, suggest that the treatment is safe and probably beneficial in carefully selected adolescents whose chronic migraine has resisted standard prophylaxis [126,127]. Botulinum toxin has also been applied to a scatter of other head and neck problems in young people, including bruxism and masseter hypertrophy, spastic trismus that limits mouth opening, and chronic sialadenitis, but for these the paediatric evidence is confined to small series and individual reports, so that treatment is offered case by case rather than as established practice [6].

Individual longitudinal cohorts are consistent with this cautious optimism, including a retrospective analysis by Shah et al. that reported a reduction in headache frequency after onabotulinumtoxinA in paediatric chronic migraine [128] and an earlier series in medically intractable paediatric chronic daily headache [129].

Evidence and clinical position. We grade the certainty as low for chronic migraine in adolescents. Two randomised trials have been performed, and they reached different conclusions, so the randomised evidence is inconsistent rather than absent, and the systematic review and meta-analysis that pool this literature rest on small samples with a high placebo response, which is characteristic of paediatric headache trials. Botulinum toxin is an option reserved for carefully selected adolescents whose chronic migraine has resisted standard prophylaxis, rather than an established preventive treatment, as it is in adults. For the remaining head and neck applications, namely bruxism and masseter hypertrophy, spastic trismus and chronic sialadenitis, the certainty is very low, the evidence being confined to small series and individual reports, and treatment is properly offered case by case.

## 8. Neonatal (Obstetric) Brachial Plexus Palsy

Traction on the brachial plexus during a difficult delivery injures the upper nerve roots in one to three of every thousand live births, and although most infants recover, a minority are left with lasting weakness. The characteristic pattern spares the strong internal rotators and adductors of the shoulder while weakening the external rotators and abductors, so that an imbalance develops which, if left unchecked, produces an internal rotation contracture and progressive deformity of the growing glenohumeral joint. A second problem is co-contraction, in which regenerating nerves become misdirected so that antagonist muscles such as biceps and triceps contract together and cancel one another during attempted movement. Botulinum toxin addresses both problems by temporarily weakening the overactive muscle, either the dominant internal rotator or the inappropriately firing antagonist, thereby restoring a more useful balance during the critical period of early motor development [130].

The landmark application was described by DeMatteo et al., who used the toxin as an adjunct to motor learning therapy and to surgery, weakening the biceps or triceps to break a disabling co-contraction [130]. Later work extended the approach to the shoulder, where injection of the internal rotators improves passive and active external rotation and can help reduce or prevent posterior subluxation, sometimes averting or deferring reconstructive surgery [131]. A systematic review and meta-analysis has since pooled the available series and confirmed measurable gains in shoulder external rotation and in joint function, while showing that the benefit is greater when the toxin is given early and is blunted in older children in whom fixed contracture and bony change have already developed. As with the other paediatric uses, the evidence rests largely on case series and small cohorts rather than randomised trials, so the toxin is best seen as one component of a coordinated programme of therapy, splinting, and, where needed, nerve or musculoskeletal surgery [132].

A number of cohort studies fill out this picture. Michaud et al., in a retrospective cohort at a dedicated brachial plexus centre, found that injection of the internal rotators or of co-contracting muscles improved shoulder external rotation and elbow function and averted, modified, or deferred surgery in a substantial proportion of children [133]. A systematic review by Gobets et al. identified four recurring indications, namely internal rotation and adduction contracture of the shoulder, limited active elbow flexion, limited active elbow extension, and forearm pronation contracture, with benefit reported for all except limited elbow extension [134]. Heise et al. specifically documented relief of the biceps and triceps co-contraction that impairs elbow control [135].

The outcome instruments used in this literature should be named because they determine how far the reported gains can be compared or pooled. Motor recovery in neonatal brachial plexus palsy is assessed principally with the Active Movement Scale, an ordinal scale developed specifically for infants that grades active movement at each joint with gravity eliminated and against gravity, and with the Mallet classification, a five-grade observational scale of functional shoulder positions that requires a cooperative child and is therefore usable from roughly three years of age. Passive and active external rotation measured in degrees, the aggregate modified Mallet score, the Toronto Test Score, and the Assisting Hand Assessment also appear in these cohorts, while glenohumeral deformity is graded on ultrasound or magnetic resonance measures of glenoid version and of posterior humeral head subluxation. Because these are ordinal rather than interval scales, because individual cohorts report different subsets of them, and because several are rated by assessors aware of the treatment given, the pooled estimates in the systematic reviews cited above should be read as indicating the direction of effect rather than its magnitude.

The observation that benefit is greater when the toxin is given early deserves expansion, because it bears directly on referral timing. No formal age threshold has been validated in this population, and we identified no study designed to determine one. The rationale for early treatment is therefore biological rather than empirical. Glenohumeral dysplasia in this condition begins within the first year of life; posterior subluxation and increasing glenoid retroversion have been demonstrated on imaging in infants within the first months, and both progress for as long as the muscle imbalance persists. Once the glenoid has remodelled and the internal rotation contracture has become fixed, weakening the internal rotators can no longer restore alignment, because the limiting structures are now bone and capsule rather than muscle activity. Most reported cohorts have accordingly treated infants and young children rather than older ones. The defensible practical position is that any child with a persisting internal rotation contracture of the shoulder or with disabling co-contraction should be referred to a specialist brachial plexus service without waiting for further spontaneous recovery, and that the expected value of botulinum toxin declines as passive external rotation becomes fixed. We recommend that future studies report outcome stratified by age at injection and by the presence or absence of established glenohumeral dysplasia so that an evidence-based threshold can eventually be derived.

Evidence and clinical position. We grade the certainty of evidence as low. No randomised controlled trial has been performed in this indication. The evidence consists of retrospective cohorts and small prospective series from specialist centres, together with two systematic reviews of that same material, and outcome assessment is generally unblinded and uses ordinal scales in a condition with substantial spontaneous recovery, a combination that will tend to overestimate the treatment effect rather than underestimate it. Botulinum toxin is positioned here as an adjunct within a coordinated programme of therapy, splinting and, where indicated, nerve or musculoskeletal surgery. Its two defensible roles are the temporary correction of muscle imbalance during the window in which glenohumeral remodelling can still be influenced, and the interruption of co-contraction to permit motor relearning. It is not an alternative to surgical reconstruction where that is indicated, and it has no established role once the internal rotation contracture has become fixed and the limiting structures are bone and capsule.

## 9. Aesthetic Use and Hyperhidrosis in Adolescents

Two further applications sit at the border between medicine and lifestyle, the treatment of excessive sweating and the aesthetic softening of facial lines. Both are common in adults, and both raise particular questions when the patient is still a minor.

### 9.1. Primary Focal Hyperhidrosis

Primary focal hyperhidrosis, excessive sweating of the axillae, palms, soles, or face without an underlying cause, typically begins in childhood and intensifies during adolescence, when it can be genuinely disabling in social and school life. Botulinum toxin injected into the dermis blocks the cholinergic fibres that drive the eccrine sweat glands, and in adults a large randomised, placebo-controlled trial established that it produces a marked and durable reduction in axillary sweating with a good safety profile [136]. The paediatric evidence is smaller but consistent. A case series in adolescents with palmar and axillary hyperhidrosis found that injection substantially improved quality of life and reduced social isolation [137], and a long-term follow-up of children and adolescents with palmar disease confirmed sustained benefit across repeated treatments [138]. The main practical obstacle in this age group is the pain of injection, particularly in the palm, which often calls for topical anaesthesia, a nerve block, or brief sedation, and the effect wears off within several months so that treatment must be repeated [137,138].

The strongest paediatric evidence comes from a multicentre open-label study by Glaser et al. in adolescents aged twelve to seventeen with severe axillary hyperhidrosis, in which onabotulinumtoxinA produced marked reductions in sweating and improved quality of life across repeated treatments, with responders typically needing only two or three treatments a year [139].

Evidence and clinical position. We grade the certainty as low to moderate, low when the paediatric evidence is considered alone and moderate once the strong adult randomised evidence for axillary disease is credited. No randomised trial has been conducted in children or adolescents, and the paediatric evidence consists of a multicentre open-label study, a case series and a long-term uncontrolled follow-up, so that the strong randomised evidence in axillary hyperhidrosis is adult evidence extrapolated to adolescents. The consistency of the paediatric results and the objective nature of the response, which can be quantified gravimetrically, make this extrapolation more secure than in most of the indications graded low in this review. Botulinum toxin is a reasonable option for adolescents with severe axillary hyperhidrosis that has not responded to topical aluminium salts, and for palmar disease it is effective but limited in practice by the pain of injection, which usually requires topical anaesthesia, a nerve block or brief sedation. Treatment must be repeated within several months.

### 9.2. Aesthetic and Cosmetic Use

Aesthetic injection to soften dynamic facial lines is the single most common use of botulinum toxin worldwide, but almost all of this activity is in adults, and its extension to people under eighteen is contentious. Genuine cosmetic indications in this age group are rare, and most authorities regard purely aesthetic treatment of a minor as inappropriate except in unusual circumstances of marked psychosocial distress, where it should follow careful counselling of the young person and the family together with a clear acknowledgement that the effect is temporary. There are, by contrast, reconstructive uses at the edge of the aesthetic field that can be justified in adolescents, such as injection to improve the appearance of a facial scar by reducing the muscular tension that widens it, or to soften a facial asymmetry, but these remain off-label and are supported only by limited evidence. The guiding principle is that the threshold for treating a child for an appearance-related concern should be considerably higher than for an adult, and that a medical rather than a purely cosmetic rationale should be present [6].

Evidence and clinical position. We grade the certainty as very low, and we note that the limiting consideration in this indication is ethical rather than evidential. No paediatric efficacy or safety data exist for purely aesthetic injection, and the practice rests entirely on extrapolation from a large adult experience. Botulinum toxin has no established place in the treatment of appearance-related concerns in people under eighteen, and purely cosmetic treatment of a minor should be regarded as inappropriate other than in unusual circumstances of marked psychosocial distress, after counselling of the young person and the family together. The narrow reconstructive exceptions, such as reducing the muscular tension that widens a facial scar or softening a facial asymmetry, are off-label, supported only by limited evidence, and should be undertaken on a medical rather than a cosmetic rationale.

## 10. Safety, Adverse Events, and Dosing

### 10.1. Adverse Events and Their Prevention

Safety in this field is best presented as a stratification rather than as a single global statement, because the risks are not comparable across the range of children treated. A fifteen-year-old receiving 50 units into the axillae for hyperhidrosis and a non-ambulant child with spastic quadriplegia, epilepsy, and bulbar dysfunction receiving 20 units per kilogram across eight muscles under general anaesthesia occupy opposite ends of a wide spectrum. We therefore separate the reported events into three groups. The first comprises common, minor, and self-limiting events, namely injection site pain, transient local swelling or bruising, excessive weakness of the injected muscle, brief lethargy or flu-like symptoms, and, after salivary gland injection, thickened secretions or dry mouth. These resolve without specific treatment and do not usually alter the decision to treat again. The second comprises uncommon events caused by local spread beyond the intended target, such as ptosis and vertical deviation after periocular injection, neck extensor weakness after injection of the sternocleidomastoid or the salivary glands, urinary retention after detrusor injection, and transient faecal incontinence after intrasphincteric injection. These are self-limiting but can be functionally significant for several weeks and should be disclosed specifically during consent for the relevant indication. The third comprises rare but serious events attributable to distant spread, namely generalised weakness, dysphagia, aspiration, and respiratory compromise, which are the basis of the boxed warning carried by every product.

The risk of events in the third group is not evenly distributed and can be anticipated to a useful degree. The recognised risk factors are low body weight, high total dose and high dose per kilogram, injection across many muscle groups within a single session, short intervals between sessions, pre-existing bulbar or respiratory compromise, an underlying disorder of neuromuscular transmission, and non-ambulant status in Gross Motor Function Classification System levels IV and V. Children with severe neurological impairment therefore carry both the greatest exposure, since they are the children most likely to receive large multilevel doses together with salivary gland injection, and the least physiological reserve with which to tolerate an adverse event. In this group we recommend that the total dose be held well below the theoretical ceiling, that salivary gland injection and large-volume limb injection not be combined in one session where this can be avoided, that swallowing be assessed formally before treatment and reviewed afterwards, and that families receive explicit written instructions on the symptoms of distant spread and on where to present urgently. New or worsening generalised weakness, dysphagia, ptosis, or respiratory difficulty appearing within days to a few weeks of injection should be treated as distant spread until proved otherwise.

Across the full range of paediatric indications, botulinum toxin has a safety record that is reassuring in aggregate, provided that the stratification set out above is applied, and the adverse events reported are predominantly local, mild, and self-limiting. In pragmatic studies that reflect everyday practice, the great majority of injection sessions in children with cerebral palsy are uneventful, and the events that do occur are mostly transient weakness, pain, lethargy, or flu-like symptoms [6,25]. Two categories of event deserve particular attention in children. The first is local spread to neighbouring muscles, producing unwanted weakness such as ptosis after periocular injection or neck weakness after injection of the sternocleidomastoid or the salivary glands. The second, and more serious, is the distant spread of toxin causing systemic weakness, difficulty swallowing, and, at the extreme, respiratory compromise, a picture resembling botulism that underlies the boxed warning carried by every product [6]. A review of adverse events reported to the United States regulator found that serious events, including the rare deaths, were far more frequent after therapeutic than after cosmetic use, a difference that reflects the higher doses and the vulnerable underlying conditions of therapeutic patients rather than a property of the toxin itself [140]. Systemic adverse events have been described specifically in children with cerebral palsy, in whom low body weight, bulbar and respiratory compromise, and the use of high doses across many muscles all increase the risk [141]. Higher doses can be given to selected children under careful monitoring, but the guiding principle is to use the lowest effective dose, to respect weight-based ceilings, and to lengthen rather than shorten the interval between sessions [142].

Immunogenicity deserves specific mention in children, who may face many years of repeated injection. Herrmann et al. showed that a minority of children treated for cerebral palsy develop antibodies to the toxin and that this can cause a secondary loss of response, more often when high doses are given at short intervals [143]. Reassuringly, the refinement of modern formulations to reduce their protein load has lowered the rate of antibody formation, so that immunogenicity with current products is low across indications [144].

Two practical points follow from this. Secondary non-response should not be attributed to antibody formation before the commoner explanations have been excluded, namely progression of the underlying condition, the development of fixed contracture in a muscle whose tone was previously dynamic, inaccurate targeting, and a dose that has become inadequate for a child who has grown since the previous session. Where immunological resistance is genuinely suspected, the appropriate responses are to lengthen the interval between sessions, to avoid booster injections within the same cycle, to use the lowest effective dose, and to consider a preparation with a lower protein load, since switching between type A products does not reliably overcome neutralising antibodies whereas a change in serotype may.

Repeated treatment over many years raises a question that is specific to childhood and that has only recently begun to be addressed, namely what cumulative chemodenervation does to a growing muscle. The concern is not theoretical. In healthy adult volunteers, a single injection into the lateral gastrocnemius produced magnetic resonance changes still present at one year, and muscle biopsy at that point showed fibre cross-sectional area reduced by about a quarter relative to a saline-injected control muscle [145]. In children with spastic cerebral palsy, three-dimensional ultrasound six months after a first injection showed a seventeen per cent reduction in mid-belly cross-sectional area once normalised for skeletal growth, together with an increase in echo-intensity consistent with a greater proportion of non-contractile tissue, changes that reached significance only in comparison with toxin-naive children, while longitudinal muscle growth was preserved [146]. A synthesis of the animal and human data has concluded that repeated injection produces atrophy with partial replacement of contractile tissue by fat and connective tissue, and has argued that clinical protocols should move towards less frequent use with closer monitoring of both benefit and harm [147]. These findings do not overturn the established role of the toxin in focal spasticity, but they argue strongly against reflexive re-injection on a fixed calendar. In practice this means preferring an interval of at least six months where the clinical situation permits, justifying each re-injection by a defined and reassessed goal rather than by routine, using the lowest effective dose in the smallest number of muscles that will achieve that goal, and recording the cumulative number of injection cycles a child has received so that it can be weighed explicitly in every subsequent decision.

The consequences for the growing skeleton are much less well characterised, and we state plainly that direct paediatric evidence is lacking. Bone accrual in childhood depends on mechanical loading by muscle, and children with cerebral palsy, particularly those in Gross Motor Function Classification System levels IV and V, already have reduced bone mineral density and a raised fracture rate for reasons that include immobility, anticonvulsant therapy, and undernutrition. Whether repeated chemodenervation adds materially to that risk has not been tested, and we identified no study using bone density or fracture incidence as an endpoint. This is a genuine gap in knowledge rather than a reassuring absence of harm, and it should be a priority for long-term follow-up.

Sedation and general anaesthesia should be counted as part of the risk of treatment rather than treated as a separate logistical matter. Most young children require pharmacological support for multilevel injection, and in pragmatic cohorts a proportion of the adverse events recorded around injection sessions have been attributable to the sedation rather than to the toxin. Children with severe neurological impairment, obstructive sleep apnoea, chronic aspiration, or a difficult airway carry the greater share of this risk, and that risk accumulates precisely because these are the children who need the most frequent sessions. Anaesthetic risk therefore belongs in the discussion with the family; it should be weighed alongside the expected duration of benefit when the re-injection interval is chosen, and it is among the strongest practical arguments for combining injection with other procedures that require anaesthesia whenever this is feasible.

### 10.2. Dosing and a Summary of Paediatric Indications

Paediatric dosing is always calculated by body weight and expressed in the units of the specific product, and because the products are not interchangeable, the figures that follow are tied to particular preparations. For focal spasticity the European consensus set widely used ceilings of the order of 20 units per kilogram up to a total of about 400 units for onabotulinumtoxinA and incobotulinumtoxinA, with correspondingly higher numerical totals for abobotulinumtoxinA, distributed across the target muscles [21]. The approved regimen for neurogenic detrusor overactivity is 200 units of onabotulinumtoxinA not exceeding 6 units per kilogram, injected into the detrusor [56], while for chronic sialorrhoea the approved incobotulinumtoxinA dose is weight banded from 20 to 75 units divided between the salivary glands [109]. Table 3 summarises the principal paediatric indications discussed in this review, the usual anatomical target, the regulatory status, and the strength of the supporting evidence.

**Table 3:** Principal paediatric indications for botulinum toxin are discussed in this review, with the usual anatomical target, regulatory status in children, and the general strength of the evidence. Approval status varies by product and country. For each indication the table also states the number and type of studies supporting it, the principal outcomes reported, the principal limitations of that evidence, the leading safety concern, and an overall certainty rating together with the clinical position of the treatment relative to available alternatives. Regulatory statements were verified against United States Food and Drug Administration prescribing information and European Medicines Agency summaries of product characteristics current at the date of the search. Approval is specific to the individual product, the age band, the dose, the indication, and the country, and an approval held by one type A product does not extend to another. Readers must confirm the status of the specific preparation against their own national labelling before use.

Indication	Usual Target	Regulatory Status	Evidence Base, Number and Type of Studies	Principal Reported Outcomes	Principal Limitations	Key Safety Concern	Certainty and Clinical Position
Cerebral palsy, lower limb spasticity	Gastrocnemius, soleus, other limb muscles	Approved. onabotulinumtoxinA and abobotulinumtoxinA, FDA and EMA, from age 2	Randomised controlled trials of onabotulinumtoxinA and abobotulinumtoxinA, including a dose-ranging trial and a comparison with serial casting, one randomised trial with 3.5-year follow-up, and one 3-year prospective controlled cohort, together with the American Academy of Neurology evidence-based assessment and practice parameter [9,77,79,80,81,82,83,84,85,86,87,88,89]	Reduced Modified Ashworth and Tardieu scores, improved gait scores and ankle kinematics. No GMFM-66 difference over 3 years in a controlled cohort	Tone-level primary endpoints, short follow-up, heterogeneous functional instruments, few trials powered for function	Distant spread at high multilevel doses. Reduced cross-sectional muscle growth after repeated injection	High for tone, low for function. First-line focal treatment, adjunct to therapy and orthoses, defers rather than prevents surgery
Cerebral palsy, upper limb spasticity	Wrist, elbow, finger flexors	Approved. onabotulinumtoxinA and abobotulinumtoxinA, FDA, from age 2	Randomised controlled trials, including pivotal trials of abobotulinumtoxinA and incobotulinumtoxinA, pooled in a meta-analysis of 14 randomised trials in 621 children appraised with RoB 2 and GRADE, together with best-practice consensus recommendations [78,90,95,99,100,101,102,103]	Wrist Ashworth mean difference −0.85. Goal Attainment Scaling improved. Melbourne Assessment and Assisting Hand Assessment not consistently improved	Divergence between subjective and objective measures. Goal Attainment Scaling not comparable across studies. Therapy co-intervention in every trial	Local weakness of grip, distant spread rare at these doses	Moderate for tone and goal attainment, low for measured function. Adjunct, only alongside occupational therapy
Functional constipation, IAS achalasia	Internal anal sphincter	Off-label, all products and jurisdictions	2 small RCTs plus retrospective cohorts and case series [26,30]	Short-term symptomatic improvement and reduced resting sphincter pressure	Variable diagnostic criteria, wide dose range, differing injection sites, follow-up often shorter than duration of effect, response independent of manometry	Transient faecal incontinence and perianal soiling	Low to moderate. Second-line reversible alternative to myectomy after optimised medical management
Persistent obstruction after Hirschsprung surgery	Internal anal sphincter	Off-label, all products and jurisdictions	Retrospective cohorts and one meta-analysis, no RCT [39,40]	Reduced obstructive symptoms and enterocolitis episodes, reduced progression to myectomy	No control group, heterogeneous surgical technique and definitions of obstruction, repeated injections commonly needed	Transient incontinence, repeated exposure to anaesthesia	Low. Early reversible step before internal sphincter myectomy
Chronic anal fissure	Internal or external anal sphincter	Off-label, all products and jurisdictions	Small paediatric series with extrapolation from adult RCTs [42,43]	Healing and pain relief comparable to topical agents in adults	Very few paediatric patients, no paediatric RCT, high spontaneous healing rate confound uncontrolled reports	Transient incontinence to flatus	Very low. Intermediate step between topical therapy and lateral internal sphincterotomy
Oesophageal achalasia	Lower oesophageal sphincter	Off-label, all products and jurisdictions	Case series only, no paediatric RCT [44,45]	Short-term relief of dysphagia, with relapse in most children within months	Small numbers, short follow-up, repeated injection may complicate subsequent myotomy	Chest pain, reflux, submucosal fibrosis	Low. Temporising bridge to dilatation or myotomy, or an option where definitive treatment is contraindicated
Neurogenic detrusor overactivity	Detrusor	Approved. onabotulinumtoxinA, FDA from age 5, 200 units not exceeding 6 units per kilogram	One adequately powered phase 3 RCT plus systematic reviews of cohorts [56]	Reduced daytime incontinence episodes, increased maximum cystometric capacity, reduced maximum detrusor pressure	Single pivotal trial, urodynamic surrogate endpoints, no renal or long-term endpoints, repeat-injection data observational	Urinary tract infection, haematuria, anaesthesia, rare generalised weakness	Moderate. Second-line after antimuscarinics and clean intermittent catheterisation, repeatable alternative to or bridge before augmentation cystoplasty
Idiopathic overactive bladder	Detrusor	Off-label, all products and jurisdictions	Retrospective and small prospective cohorts, no paediatric RCT [67,68]	Improved continence and reduced urgency in children refractory to antimuscarinics	No control group, variable definitions, short follow-up, spontaneous improvement common at this age	Urinary retention requiring catheterisation, urinary tract infection	Low. Third-line, after behavioural therapy and pharmacotherapy have failed
Detrusor sphincter dyssynergia, dysfunctional voiding	External urethral sphincter	Off-label, all products and jurisdictions	Small case series only [71]	Reduced post-void residual volume and improved flow in selected children	Very small numbers, no controls, substantial overlap with behavioural factors	Transient stress incontinence	Very low. Adjunct that opens a window for biofeedback and pelvic floor retraining
Sialorrhoea	Parotid and submandibular glands	Approved. incobotulinumtoxinA, FDA from age 2, weight-banded 20 to 75 units. Other products off-label	One phase 3 RCT (SIPEXI) with earlier randomised trials and cohorts [109]	Reduced unstimulated salivary flow rate and improved global impression of change	Salivary flow is a surrogate for the burden of drooling, blinded functional and quality-of-life endpoints are less consistent	Thickened secretions, dry mouth, dysphagia and aspiration in children with bulbar impairment	High. Principal pharmacological option where anticholinergics fail or are not tolerated, alternative to salivary gland surgery
Congenital muscular torticollis	Sternocleidomastoid, upper trapezius	Off-label, all products and jurisdictions	Case series and one combined protocol study, no RCT [116,117]	Improved cervical rotation and head tilt, fewer children proceeding to surgery	No RCT, no blinding, high spontaneous and physiotherapy-related recovery rate	Neck extensor weakness, transient dysphagia	Very low. Adjunct in physiotherapy-resistant cases before surgery is considered
Strabismus	Extraocular muscles	Approved. onabotulinumtoxinA, FDA, from age 12. Use below age 12 is off-label	One Cochrane systematic review, two randomised trials comparing injection with reoperation in 47 and 55 children, and two retrospective non-randomised comparative studies including a three-year multicentre noninferiority study in 76 children [120,122,123,124,125]	Reduction in horizontal deviation in prism dioptres, with motor alignment comparable to surgery in selected squints. The randomised comparisons with reoperation found no significant difference in motor or sensory outcome. In the three-year noninferiority study success was 72 per cent after toxin and 56 per cent after surgery, with similar median stereoacuity and a shorter duration of general anaesthesia	Low certainty in the Cochrane assessment, sensory outcomes reported inconsistently, higher reintervention rate than surgery	Transient ptosis, induced vertical deviation	Low overall and moderate in acute acquired comitant esotropia. First-line alternative to surgery in small and moderate angle esotropia, adjunct in residual deviation
Dystonia	Focal target muscles	Off-label. Blepharospasm approved for onabotulinumtoxinA from age 12	Two systematic reviews, both of which identified only open-label series and case reports with no controlled paediatric trials [104,105]	Reduced pain, easier positioning, easier delivery of care	No control group, no blinding, outcome measures not standardised	Excessive weakness in already weak children, dysphagia after cervical injection	Very low. Symptom-directed adjunct for the most disabling focal component, not a treatment for generalised dystonia
Neonatal brachial plexus palsy	Shoulder internal rotators, co-contracting muscles	Off-label, all products and jurisdictions	Retrospective cohorts, small prospective series and 2 systematic reviews, no RCT [130,132]	Improved active and passive shoulder external rotation and elbow control, surgery averted or deferred in a proportion	Unblinded assessment in a condition with substantial spontaneous recovery, ordinal scales, no validated age threshold	Transient excessive weakness of the injected muscle	Low. Adjunct within a therapy, splinting and surgical programme, most useful before glenohumeral dysplasia is established
Chronic migraine, adolescents	Fixed head and neck protocol sites	Off-label. onabotulinumtoxinA approved for chronic migraine from age 18 only	Two systematic reviews, one with meta-analysis, covering two small randomised trials that reached discordant conclusions together with predominantly observational data [126,127]	Reduced headache days and improved PedMIDAS disability scores in selected adolescents	Discordant randomised evidence, strong placebo response in adolescent headache, heterogeneous definitions of refractoriness	Neck weakness, injection-site pain, ptosis	Low. Reserved for adolescents whose chronic migraine has failed standard prophylaxis
Primary focal hyperhidrosis	Intradermal axilla or palm	Off-label in children. onabotulinumtoxinA approved for primary axillary hyperhidrosis from age 18	One multicentre open-label paediatric study and case series, with a large adult RCT [137,138]	Marked reduction in sweating and improved quality of life, typically 2 to 3 treatments a year	Open-label paediatric data, no paediatric RCT, subjective primary endpoints	Injection pain requiring anaesthesia, transient intrinsic hand weakness after palmar injection	Low to moderate. Second-line after topical antiperspirants and iontophoresis, before systemic anticholinergics or sympathectomy
Posterior urethral valve bladder	Detrusor or external sphincter	Off-label, all products and jurisdictions	One small randomised controlled trial, 20 boys	No difference from control in infection, hydronephrosis, reflux, creatinine, cystometric capacity or voiding pressure	Only 20 boys, underpowered, follow-up of 6 months, no long-term renal outcomes	Urinary tract infection, retention	Low. Bladder neck injection not supported by the only randomised evidence, intradetrusor use unproven and confined to structured urodynamic surveillance

Approved indications reflect regulatory decisions in major jurisdictions such as the United States and the European Union and do not imply approval of a specific product in every country. Off-label denotes use supported by published evidence but without a specific paediatric marketing authorisation. RCT, randomised controlled trial. IAS, internal anal sphincter.

## 11. Limitations and Future Directions

Several themes recur across the indications reviewed here. The first is the scarcity of high-quality evidence. With the notable exceptions of cerebral palsy, spasticity, neurogenic detrusor overactivity, and chronic sialorrhoea, most paediatric uses rest on open-label series, small cohorts, and individual reports rather than randomised controlled trials, and the studies that do exist are often heterogeneous in dose, technique, outcome measure, and length of follow-up. This heterogeneity makes formal comparison difficult and leaves many recommendations dependent on expert consensus and on extrapolation from adult practice [6].

One limitation of this review itself should be restated here rather than left in the Methods, because it bears directly on how the organ system sections should be read. We deliberately included case series and uncontrolled reports, since applying a minimum methodological threshold would have left several indications with no evidence at all and would have misrepresented current practice. The consequence is that a substantial proportion of the studies cited above carry a high risk of bias, that uncontrolled response rates are likely to overestimate the true effect in conditions with appreciable spontaneous improvement such as functional constipation, congenital muscular torticollis, and neonatal brachial plexus palsy, and that the reader should weight every statement in this review by the certainty rating attached to it rather than by the confidence of its phrasing. A second limitation of our own method should be recorded alongside it. Eligibility was restricted to English-language reports, which risks omitting relevant paediatric experience published in other languages, particularly from centres in continental Europe, Asia, and Latin America where botulinum toxin is used widely in children. We cannot quantify the effect of this restriction, and it should be borne in mind wherever we describe the paediatric evidence for an indication as scarce.

A second theme is the persistence of questions that are specific to childhood. The long-term consequences of repeated chemodenervation on the growth of muscle and bone are incompletely understood, the optimal timing and frequency of injection are rarely defined, and dosing in the youngest children, particularly those under two years of age, remains largely off-label and guided by cautious extrapolation. The immature neuromuscular junction may respond differently from that of the adult, and the balance between an early therapeutic window and possible effects on a developing system has not been fully resolved [6].

Future progress is likely to come from several directions. Ultrasound and other imaging guidance are improving the accuracy of injection and may reduce both the dose required and the risk of adverse effects, particularly for deep muscles and for glands. Standardised, child-specific outcome measures and agreed core outcome sets would allow trials to be compared and pooled. Longer-acting preparations may reduce the frequency of injection and the associated burden of sedation, and the growing understanding of the sensory and autonomic actions of the toxin, beyond simple motor blockade, may open new indications in pain and in glandular and visceral disorders. Above all, adequately powered randomised trials with functional and patient-centred endpoints are needed to move several promising but unproven paediatric applications onto a firmer evidence base [6].

Two further recommendations follow directly from the concerns discussed above. The first is that future paediatric spasticity trials should report standardised motor and functional outcome measures and should state explicitly at which level of the International Classification of Functioning, Disability and Health each endpoint sits. A trial reporting only the Modified Ashworth Scale answers a question about tone and cannot answer a question about walking, and the common practice of powering a trial for a tone endpoint while discussing it in functional language is the single most important source of overstatement in this literature. Agreement on a core outcome set that pairs a tone measure with a validated activity measure such as the Gross Motor Function Measure and with a participation measure would allow trials to be pooled rather than merely listed. The second is that long-term studies should report the cumulative number of injection cycles, the interval between them, and objective measures of muscle morphology together with, where feasible, bone density, since these are the paediatric-specific harms about which the evidence is currently weakest.

## 12. Conclusions

Botulinum toxin has travelled a remarkable path from one of the most feared natural poisons to a precise and versatile paediatric therapeutic, yet the evidence behind its expanding use remains markedly uneven. Relatively strong support exists for only a few indications. Focal spasticity of the lower and upper limb in cerebral palsy is backed by multiple randomised controlled trials and meta-analyses, although that strength attaches to the reduction in muscle tone rather than to gross motor function, gait, or participation. Neurogenic detrusor overactivity is supported by an adequately powered randomised trial and by regulatory approval from five years of age, with fewer incontinence episodes and improved urodynamic parameters as the demonstrated outcomes. Chronic sialorrhoea is supported by randomised evidence, including a paediatric phase three trial, and esotropia of small to moderate angle, including the acute acquired comitant form, by randomised comparison with surgery for motor alignment.

The majority of indications rest on uncontrolled cohorts and case series, frequently without blinding and often in conditions in which spontaneous improvement is common. These include functional constipation and internal anal sphincter dysfunction, persistent obstruction after surgery for Hirschsprung disease, chronic anal fissure, oesophageal achalasia, idiopathic overactive bladder, detrusor sphincter dyssynergia, the valve bladder, congenital muscular torticollis, childhood dystonia, neonatal brachial plexus palsy, adolescent chronic migraine, and primary focal hyperhidrosis. For these, botulinum toxin is best described as a reasonable option in selected refractory patients rather than as an established treatment, and references in earlier sections to protecting the kidneys or restoring continence should be read strictly as shorthand for reduced bladder storage pressure and fewer incontinence episodes, since no paediatric study has used preservation of renal function or durable continence as an endpoint. Two qualifications apply across the whole field. Most paediatric use remains off-label, with approval specific to the product, age band, dose, indication, and country, so a large part of practice rests on consensus statements and extrapolation from adult data. And although the aggregate safety record is genuinely good, serious adverse events cluster in children of low body weight, in those with bulbar or respiratory compromise, in those receiving high multilevel doses at short intervals, and in the non-ambulant, while the consequences of cumulative chemodenervation for the growing muscle and skeleton remain incompletely characterised.

The research priorities follow directly. Whether tone reduction in cerebral palsy translates into durable gains in function and participation and in which children, what injection interval is optimal once growth of the treated muscle is taken into account, whether repeated treatment affects bone density and fracture risk in non-ambulant children, and whether the off-label gastrointestinal and urological uses alter the natural history of disease or only its symptoms all remain unanswered. Until adequately powered randomised trials with functional and patient-centred endpoints address these questions, botulinum toxin is best regarded as a reversible, repeatable, and generally well-tolerated adjunct whose place differs sharply between indications and should be chosen according to the certainty ratings rather than by analogy from one indication to another.

## Data Availability

No new data were created or analysed in this study. Data sharing is not applicable to this article.

## Supplementary Materials

The following supporting information can be downloaded at https://www.mdpi.com/article/10.3390/toxins18090360/s1, Table S1: Study-level appraisal of methodological quality and risk of bias for the studies underpinning the certainty rating of each indication; Table S2: Certainty rating, supporting references, and risk of bias by indication, summarised from Table S1.

## Abbreviations

The following abbreviations are used in this manuscript:

AMSTAR-2	A MeaSurement Tool to Assess Systematic Reviews, Version 2
CI	Confidence interval
EMA	European Medicines Agency
FDA	United States Food and Drug Administration
GMFCS	Gross Motor Function Classification System
GMFM-66	Gross Motor Function Measure, 66-item version
GRADE	Grading of Recommendations, Assessment, Development and Evaluations
IAS	Internal Anal Sphincter
ICF	International Classification of Functioning, Disability and Health
MEDLINE	Medical Literature Analysis and Retrieval System Online
PEDI	Pediatric Evaluation of Disability Inventory
PedMIDAS	Pediatric Migraine Disability Assessment Score
PRISMA	Preferred Reporting Items for Systematic Reviews and Meta-Analyses
RCT	Randomised Controlled Trial
RoB 2	Revised Cochrane Risk-of-Bias Tool for Randomised Trials, Version 2
ROBINS-I	Risk Of Bias In Non-randomised Studies of Interventions
SIPEXI	Sialorrhoea Pediatric Xeomin Investigation Trial
