# Supplementary material for: Botulinum Toxin in Children and Adolescents—A Comprehensive Review of Clinical Applications
Source: Toxins (Basel). 2026 Aug 23;18(9):360. doi: 10.3390/toxins18090360 (PMC13611330; doi:10.3390/toxins18090360)
Supplement: Supplementary file 1 [file toxins-18-00360-s001.zip › toxins-4473761-supplementary.pdf]

Supplementary Materials. Botulinum Toxin in Children and Adolescents, A Comprehensive Review of Clinical Applications

Table S1. Study-level appraisal of methodological quality and risk of bias for the studies underpinning the certainty rating of each indication.

The appraisal instrument was matched to design, with the revised Cochrane tool for randomised trials, ROBINS-I for nonrandomised comparative studies and cohorts, the Joanna Briggs Institute checklist for case series and uncontrolled reports, and AMSTAR-2 for systematic reviews and meta-analyses. Guidelines, evidence-based assessments and consensus statements are cited as sources of recommendation and were not appraised with a study-level instrument. Judgements were made from the published report, information absent from a report is recorded explicitly rather than inferred, and studies whose full text was not obtainable are marked as not appraised and carry no judgement.

Reference and study	Indication	Design	Instrument	Domain-level judgements	Overall judgement	Notes bearing on interpretation
Delgado et al. 2016 [79]	Cerebral palsy, lower limb spasticity	Phase 3 double-blind randomised placebo-controlled single-dose trial, 241 randomised and 226 completed, ages 2 to 17, placebo comparator.	RoB 2	D1 low, D2 low, D3 low, D4 low, D5 low	Low risk	The primary endpoint was muscle tone and no standardised functional instrument was used, so the functional endpoints were clinician impression and individualised goal attainment.
Russo et al. 2007 [101]	Cerebral palsy, upper limb spasticity	Single-blind randomised controlled trial, 43 children with hemiplegic cerebral palsy aged 3 to 16, injection plus occupational therapy versus occupational therapy alone.	RoB 2	D1 low, D2 some concerns, D3 some concerns, D4 high for tone outcomes and low for activity and quality-of-life outcomes, D5 low	Some concerns overall, high risk for the tone outcomes	Outcomes that improved were unblinded or child-reported, while every blinded functional measure showed no difference, a clear tone versus function gap.
Koman et al. 1994 [80]	Cerebral palsy, lower limb spasticity	Preliminary double-blind randomised placebo-controlled trial, 12 ambulatory children aged 4 to 11, six toxin and six placebo, placebo comparator. Full text not available for re-verification, and the design details are corroborated from the descriptions in [83,86].	RoB 2	D1 some concerns, D2 high, D3 some concerns, D4 some concerns, D5 high	High risk	A pilot double-blind trial of 12 ambulatory children, six per arm, with five of six toxin-treated children improving on a physician rating scale, so the trial establishes direction of effect only and is far too small to estimate an effect size.
Koman et al. 2000 [81]	Cerebral palsy, lower limb spasticity	Randomised double-blind placebo-controlled trial of lower limb injection. Full text not obtainable, so no further design detail is available.	Not appraised	Not appraised, full text not obtainable	Not appraised, full text not obtainable.	Counted among the primary lower limb studies in Table S2, with no full text available at the time of appraisal.
Dabrowski et al. 2021 [99]	Cerebral palsy, upper limb spasticity	Double-blind randomised parallel-group phase 3 trial, 351 randomised, ages 2 to 17, comparator low-dose 2 units per kilogram rather than placebo.	RoB 2	D1 low, D2 low in the double-blind period and high in the open-label extension, D3 low, D4 low for Ashworth and some concerns for global impression, D5 low	Some concerns for the double-blind main period and high risk for the open-label extension	There was no placebo arm, so the trial establishes dose–response rather than efficacy, and only the first of four cycles was blinded.
Olesch et al. 2010 [102]	Cerebral palsy, upper limb spasticity	Randomised controlled trial of three injection cycles with occupational therapy, 24 enrolled and 22 analysed, young children with hemiplegia, occupational therapy comparator.	RoB 2	D1 some concerns, D2 high, D3 some concerns, D4 high, D5 low	High risk	The primary outcomes that improved were unblinded parental perception, while the observer-administered movement quality and fine motor measures showed no improvement.
Sutherland et al. 1999 [83]	Cerebral palsy, lower limb spasticity	Randomised double-blind parallel-group trial, 20 enrolled and 19 analysed, ages 2.4 to 12.5 years, saline vehicle placebo comparator.	RoB 2	D1 some concerns, D2 low, D3 low, D4 low for kinematics and some concerns for electromyography, D5 some concerns	Some concerns	Significant effects were confined to ankle joint angle, with no change in step length, stride length, cadence or walking velocity.

Reddihough et al. 2002 [86]	Cerebral palsy, lower limb spasticity	Randomised crossover trial over 12 months without placebo, 61 recruited and 49 analysed, ages 22 to 80 months, physiotherapy alone comparator.	RoB 2, crossover variant	D1 some concerns, carryover and period effects high, D2 high, D3 some concerns, D4 low for Gross Motor Function Measure and Vulpe and high for the parental questionnaire, D5 high	High risk	No Gross Motor Function Measure comparison differed between phases, yet unblinded parents rated benefit highly, repeating the tone versus function gap.
Tedroff et al. 2010 [88]	Cerebral palsy, lower limb spasticity	Randomised controlled study without placebo, 16 eligible and 15 completed, mean age 16 months, comparator a daily stretching programme alone.	RoB 2	D1 some concerns, D2 high, D3 some concerns, D4 high for tone and range of motion and low for gait analysis, D5 some concerns	High risk	Positive findings came from unblinded tone and joint range assessments, while the blinded gait analysis and functional measures showed no group difference.
Baker et al. 2002 [82]	Cerebral palsy, lower limb spasticity	Randomised double-blind placebo-controlled dose-ranging trial, 126 recruited, 125 analysed, 124 completed the 16-week assessment, ages 2 to 9, placebo comparator.	RoB 2	D1 low, D2 low, D3 low, D4 low for the primary outcome and some concerns for subjective ratings, D5 low	Low risk	The primary outcome was physiological, and the only standardised functional instrument showed no difference from placebo despite blinding maintained throughout.
Ubhi et al. 2000 [84]	Cerebral palsy, lower limb spasticity	Randomised double-blind placebo-controlled parallel trial, 40 randomised, 22 toxin and 18 placebo, ages 2 to 16, matched placebo comparator.	RoB 2	D1 some concerns, D2 some concerns, D3 low for the primary outcome and some concerns for secondary measures, D4 low, D5 some concerns	Some concerns	The primary outcome was an ordinal foot contact grading, and the physiological cost index and passive dorsiflexion were both unchanged.
Corry et al. 1998 [85]	Cerebral palsy, lower limb spasticity	Randomised prospective trial against serial casting, 20 children. Design and sample size taken from the published abstract because no full text was obtainable.	Not appraised	Not appraised, full text not obtainable	Not appraised, full text not obtainable.	Similar efficacy to serial casting with more prolonged improvement in passive dorsiflexion, and no full text was obtainable.
Scholtes et al. 2006 [87]	Cerebral palsy, lower limb spasticity	Randomised trial with a multiple baseline design, 47 randomised and 46 analysed, mean age 8.0 years, comparator usual physiotherapy on a waiting list.	RoB 2	D1 low, D2 high, D3 low for the trial analysis and some concerns at 48 weeks, D4 high for GMFM-66 and the problem score and low for energy cost, D5 low	High risk	The intervention was injection plus intensive rehabilitation against usual physiotherapy at a third of that intensity, so the trial estimates a package effect rather than the toxin.
Corry et al. 1997 [100]	Cerebral palsy, upper limb spasticity	Randomised double-blind placebo-controlled trial, 14 children, mean age 9 years with a range of 4 to 19, normal saline placebo comparator.	RoB 2	D1 some concerns, D2 high, D3 low, D4 some concerns, D5 high	High risk	Effects were mainly on tone and stiffness, while coin transfer, the one fine motor measure, was not significant at either timepoint.
Gresits et al. 2025 [90]	Cerebral palsy, upper limb spasticity	Systematic review with random effects meta-analysis of randomised controlled trials, 14 trials and 621 patients included.	AMSTAR-2	Critical and non-critical domains met except publication bias, which was not assessed	Low confidence, driven solely by the unmet publication bias domain	Reported a wrist Ashworth reduction of 0.85 with no significant functional gain, a divergence between subjective and objective results.
Delgado et al. 2021 [95]	Cerebral palsy, upper limb spasticity	Double-blind randomised repeat-treatment phase 3 study, 210 children treated in cycle 1, ages 2 to 17, comparator low-dose 2 units per kilogram active control.	RoB 2	D1 low, D2 low, D3 low, D4 low, D5 low	Low risk for the double-blind cycle 1 comparison, high risk for cycles 2 to 4 in which all children received an active dose	The comparator is a low-dose active control rather than placebo, so the trial establishes a dose response and not efficacy against no treatment.
Kawamura et al. 2007 [103]	Cerebral palsy, upper limb spasticity	Double-blind randomised trial of low against high dose upper extremity injection, 39 children, mean age 6 years 2 months, active dose comparator.	RoB 2	D1 low, D2 low, D3 low, D4 low, D5 some concerns	Some concerns	A negative trial with complete follow-up, no difference between doses in upper extremity function, and no untreated control arm.
Fehlings et al. 2026 [89]	Cerebral palsy, lower limb spasticity	Prospective observational cohort, 124 children aged 2 to 6 in GMFCS I to III, 65 injected and 59 comparison, membership by clinical decision.	ROBINS-I	Confounding serious, selection moderate, classification low, deviations moderate, missing data low, measurement moderate, reporting low	Serious risk of bias, driven by confounding	The GMFM-66 null is robust, but the range-of-motion difference is of the same order as the baseline

						imbalance and should not be read as harm.
Tejedor and Rodriguez 1998 [123]	Strabismus	Randomised controlled trial of early retreatment of infantile esotropia, 47 children, 24 surgery and 23 toxin, reoperation comparator, one-year minimum follow-up.	RoB 2	D1 high, D2 some concerns, D3 low, D4 some concerns, D5 low	High risk	No significant difference in motor or sensory outcome between reoperation and toxin, with follow-up averaging about three years.
Tejedor and Rodriguez 1999 [124]	Strabismus	Randomised controlled trial of retreatment after surgery for acquired esotropia, 55 children, 28 surgery and 27 toxin, six months minimum follow-up.	RoB 2	D1 high, D2 some concerns, D3 low, D4 some concerns, D5 low	High risk	Success reached 66.7 per cent after toxin against 75 per cent after surgery, with transient ptosis in over half of injected children.
Wan et al. 2017 [122]	Strabismus	Single-centre retrospective non-randomised comparison of 49 children with acute-onset comitant esotropia, 16 with toxin and 33 with incisional surgery, allocation by physician preference.	ROBINS-I	Confounding serious, selection moderate, classification low, deviations low, missing data moderate, measurement moderate, reporting moderate	Serious risk of bias, driven by confounding	Noninferior at six months with median stereoacuity not significantly different at six or eighteen months, and it shares authors with [125], so count them once.
Nguyen et al. 2025 [125]	Strabismus	Multicentre retrospective non-randomised noninferiority comparison over three years, 76 children. Design and sample size from the published abstract, no full text obtainable.	Not appraised	Not appraised, full text not obtainable	Not appraised, full text not obtainable.	The three-year multicentre follow-up of the same comparison, success 72 against 56 per cent, whose full text was not obtainable.
Rowe and Noonan 2017 [120]	Strabismus	Cochrane systematic review of randomised trials in strabismus: six trials eligible, too heterogeneous to pool, descriptive synthesis graded with GRADE.	AMSTAR-2	Critical and non-critical domains met except publication bias, which could not be examined	Low confidence, driven solely by the unmet publication bias domain	Found low-certainty evidence overall, with publication bias unassessed because there were too few trials for a funnel plot.
Keshtgar et al. 2007 [26]	Functional constipation and internal anal sphincter dysfunction	Double-blind randomised trial, 42 children aged 4 to 16, 21 per arm, comparator internal anal sphincter myectomy.	RoB 2	D1 some concerns, D2 low, D3 low, D4 low, D5 some concerns	Some concerns	A validated symptom severity score at three months and on longer-term follow-up underpins the comparison with myectomy.
Friedmacher and Puri 2012 [30]	Functional constipation and internal anal sphincter dysfunction	Meta-analysis comparing posterior internal anal sphincter myectomy with intrasphincteric injection for internal anal sphincter achalasia.	AMSTAR-2	Critical domains unmet for protocol registration, risk of bias appraisal and publication bias, other domains adequately reported	Critically low confidence	Three critical domains are unmet, most seriously that the included studies were never appraised for risk of bias.
Church et al. 2017 [39]	Persistent obstruction after surgery for Hirschsprung disease	Retrospective single-centre review, 12 patients, 13 ultrasound-guided against 17 unguided intrasphincteric procedures, comparator injection without ultrasound guidance.	ROBINS-I	confounding serious, selection serious, classification low, deviations low, missing data moderate, measurement moderate, reporting moderate	Serious risk of bias	Five of the twelve patients contribute to both groups, so the groups are not independent and the rating should be read as serious at best.
Roorda et al. 2019 [40]	Persistent obstruction after surgery for Hirschsprung disease	Systematic review and meta-analysis of injection after surgery for Hirschsprung disease: 14 studies included, duplicate eligibility assessment.	AMSTAR-2	Critical domains met except for protocol registration, non-critical domains met	Low confidence	One critical domain is unmet, the absence of a registered protocol, while appraisal of included studies and publication bias assessment are met.
Husberg et al. 2009 [42]	Chronic anal fissure	Uncontrolled prospective pilot case series, 13 children aged 1 to 10 years, mean 4 years, no comparator.	JBI checklist for case series	7 of 10 criteria met, no consecutive inclusion documented, no blinding, outcome not measured with a validated instrument	High risk	Explicitly presented by its authors as a pilot study, which is how it should be read.
Keshtgar et al. 2009 [43]	Chronic anal fissure	A prospective uncontrolled cohort of 16 children given 200 units transcutaneously, with a comparator of	ROBINS-I	confounding critical, selection serious, classification low, deviations moderate, missing data low, measurement moderate, reporting moderate	Critical risk of bias for the comparative element, and the uncontrolled element	Only 4 of the 16 children had a fissure and the injection was into the external sphincter, so the study does not test the stated mechanism.

		31 historical controls, assessed at 3 and 12 months.			should be read as a case series	
Ip et al. 2000 [45]	Oesophageal achalasia	Retrospective single-centre case series of 7 children with manometrically confirmed achalasia, no comparator.	JBI checklist for case series	5 of 10 criteria met, retrospective ascertainment, no blinding, locally devised outcome score, follow-up not uniform	High risk	The second achalasia study, Hurwitz 2000 [44], is not in the set supplied, so this indication currently rests on one appraised case series of 7 children.
Austin et al. 2021 [56]	Neurogenic detrusor overactivity	Multicentre randomised double-blind trial over 48 weeks, 113 children aged 5 to 17, comparator a 50-unit dose in lieu of placebo.	RoB 2	D1 low, D2 low, D3 some concerns, D4 low, D5 low	Some concerns	The 50-unit arm replaced a placebo control, so the trial compares doses and does not establish efficacy against no treatment.
Hoebeke et al. 2006 [67]	Idiopathic overactive bladder	Prospective uncontrolled series of 21 children given a single 100-unit intradetrusor injection, 15 analysed for longer-term response, no comparator.	JBI checklist for case series	7 of 10 criteria met, no blinding, response partly defined subjectively, efficacy analysis resting on 15 of 21 children	High risk	Described by its authors as a pilot study establishing safety and long-term efficacy.
Ringoir et al. 2020 [68]	Idiopathic overactive bladder	Retrospective single-centre study of intradetrusor injection of 100 units in 257 children, no comparator group.	ROBINS-I	confounding serious, selection moderate, classification low, deviations moderate, missing data moderate, measurement moderate, reporting moderate	Serious risk of bias	A retrospective single-arm series of 257 children, which is its value and also its limitation, since there is no comparator.
Steinhardt et al. 1997 [71]	Detrusor sphincter dyssynergia and dysfunctional voiding	Single case report of one 7-year-old girl with urodynamically confirmed detrusor sphincter dyssynergia, no comparator.	JBI checklist for case reports	5 of 8 criteria met, a single patient, no comparator, no blinding, outcome reported narratively	High risk, case report	A report of one child rather than a series, correctly described in the manuscript as the first application in a neurologically normal child.
Mokhless et al. 2014 [76]	Posterior urethral valve bladder	Randomised trial in 20 boys of mean age 16 months, 10 injected and 10 cystourethroscopy alone, comparator cystourethroscopy without injection.	RoB 2	D1 some concerns, D2 some concerns, D3 low, D4 some concerns, D5 some concerns	Some concerns, with serious imprecision	A negative trial with no difference on any outcome, the authors concluding that abolishing bladder neck function does not improve outcome.
Reid et al. 2008 [108]	Sialorrhoea	Not appraised; full text not obtainable.	Not appraised	Not appraised, full text not obtainable	Not appraised, full text not obtainable.	Cited in the manuscript and retained here so that the omission is declared rather than silent.
Berweck et al. 2021 [109]	Sialorrhoea	Phase 3 double-blind placebo-controlled trial, 220 randomised aged 6 to 17, 148 toxin and 72 placebo, plus 35 uncontrolled patients aged 2 to 5.	RoB 2	D1 low, D2 low, D3 low, D4 low for salivary flow rate and some concerns for carer rated global impression, D5 low	Low risk for the placebo-controlled main period, high risk for the open-label extension and for the 2 to 5 year cohort which had no control	The absence of a placebo comparator in the 2-to-5-year cohort is why both the approval and the evidence stop at 6 years.
Hung et al. 2021 [110]	Sialorrhoea	Systematic review and meta-analysis, 21 trials, 827 participants, of whom 573 had cerebral palsy, ages 1.6 to 21 years, 5 randomised and 16 observational.	AMSTAR-2	PROSPERO registration met, four-database search to January 2020 met, duplicate screening met, quality appraisal inadequate because Jadad and Newcastle-Ottawa are not domain-based, publication bias not assessed, random-effects pooling appropriate	Low confidence	Only four of the 21 trials were poolable, so the review supports the direction of effect more securely than its magnitude.
Bekkers et al. 2019 [113]	Sialorrhoea	Randomised partly single-blinded trial of submandibular injection against two-duct ligation, 57 randomised, 53 analysed by intention to treat, mean age 11 years.	RoB 2	D1 low, D2 some concerns, D3 low, D4 some concerns, D5 low	Some concerns	The only head-to-head randomised comparison against surgery, and it favoured duct ligation at 32 weeks, 63.0 against 26.9 per cent.
Alrefai et al. 2009 [114]	Sialorrhoea	Double-blind placebo-controlled trial of intraparotid Dysport: 24 children aged	RoB 2	D1 some concerns, D2 low, D3 high, D4 some concerns, D5 some concerns	High risk of bias	A small parotid-only trial contributing direction of effect rather than effect size, with

		21 months to 7 years, 100 units then 140 units at four months.				significant reductions in frequency, severity and total score.
Joyce and de Chalain 2005 [116]	Congenital muscular torticollis	Retrospective analysis of 15 children with recalcitrant infantile muscular torticollis treated by injection with physiotherapy, no comparator.	JBI checklist for case series	7 of 10 criteria met, retrospective ascertainment, no control group, outcome partly a clinical decision, outcome data independently obtained	High risk	Congenital muscular torticollis resolves with physiotherapy in most infants, so an uncontrolled avoided-surgery rate cannot separate treatment effect from natural history and continued stretching.
Sinn et al. 2023 [117]	Congenital muscular torticollis	Retrospective study of 134 of 291 reviewed infants with refractory congenital muscular torticollis, no comparator.	ROBINS-I	confounding serious, selection moderate, classification low, deviations moderate, missing data moderate, measurement moderate, reporting moderate	Serious risk of bias	A retrospective single-arm series of 134 infants, subject to the same natural history problem as [116], since the condition resolves with physiotherapy in most infants.
Bohn et al. 2021 [105]	Dystonia	Systematic review update with meta-analysis of interventions for dystonia in cerebral palsy, with registered protocol, duplicate screening and GRADE grading.	AMSTAR-2	All critical and non-critical domains met, including protocol registration and publication bias assessment	High confidence	Meets every AMSTAR-2 critical domain, so the very low certainty for dystonia reflects a weak underlying literature rather than a weak review.
DeMatteo et al. 2006 [130]	Neonatal brachial plexus palsy	Prospective single-centre cohort without a control group, 23 children with incomplete recovery after obstetric brachial plexus injury.	JBI checklist for case series	6 of 10 criteria met, no control group, no blinding, selection by clinical indication rather than consecutively, outcomes assessed by the treating team	High risk	The paper itself states that no prospective randomised controlled trials exist evaluating surgical or rehabilitative intervention after obstetric brachial plexus injury, which remains true and supports the low certainty rating.
Chen et al. 2022 [132]	Neonatal brachial plexus palsy	Systematic review and meta-analysis of botulinum neurotoxin in obstetric brachial plexus injury, 13 included articles, duplicate screening.	AMSTAR-2	Critical domains met except protocol registration, non-critical domains met	Low confidence	One critical domain is unmet, the absence of a registered protocol, and the review is otherwise well conducted.
Lindsay et al. 2024 [126]	Chronic migraine in adolescents	Systematic review and meta-analysis of onabotulinumtoxinA for chronic migraine in children and adolescents, with duplicate screening and extraction.	AMSTAR-2	Critical domains met except protocol registration, non-critical domains met	Low confidence	Well conducted apart from the missing registered protocol, with graded risk of bias, random effects pooling and publication bias testing.
Mavridi et al. 2024 [127]	Chronic migraine in adolescents	Systematic review without meta-analysis, 14 studies included, of which only 2 were randomised controlled trials, doses 20 to 200 units.	AMSTAR-2	Critical domains unmet for protocol registration, risk of bias appraisal and publication bias, other domains adequately reported	Critically low confidence	Three critical domains are unmet, most seriously that the included studies were never appraised for risk of bias.
Kouris et al. 2015 [137]	Primary focal hyperhidrosis	Prospective case series of 35 adolescents aged 13 to 16 with palmar and axillary hyperhidrosis, no comparator.	JBI checklist for case series	7 of 10 criteria met, no control group, no blinding, single centre, severity and quality of life measured with validated instruments	High risk	A prospective series of 35 adolescents, using the Hyperhidrosis Disease Severity Scale and the Children's Dermatology Life Quality Index as validated measures of severity and quality of life.
Coutinho dos Santos et al. 2009 [138]	Primary focal hyperhidrosis	Long-term uncontrolled follow-up of nine children and adolescents with palmar hyperhidrosis, no comparator.	JBI checklist for case series	5 of 10 criteria met, no control group, no blinding, sample of nine, outcomes reported narratively without a validated instrument	High risk	Its value is the duration of follow-up rather than the strength of its design.
Simpson et al. 2008 [9]	Cerebral palsy, both limbs	American Academy of Neurology evidence-based assessment.	Not appraised	Not appraised with a study-level instrument. Cited as a source of recommendation rather than as primary evidence	Not applicable	Cited as a source of recommendation, its conclusion that the toxin reduces spasticity while the functional effect is less

						certain anchors the tone versus function distinction.
Delgado et al. 2010 [77]	Cerebral palsy, both limbs	American Academy of Neurology practice parameter.	Not appraised	Not appraised with a study-level instrument. Cited as a source of recommendation rather than as primary evidence	Not applicable	Cited as a source of recommendation on dosing and patient selection, adding no independent data beyond the trials it summarises.
Strobl et al. 2015 [78]	Cerebral palsy, upper limb spasticity	Expert best-practice consensus statement.	Not appraised	Not appraised with a study-level instrument. Cited as a source of recommendation rather than as primary evidence	Not applicable	Cited as a practice recommendation, its consensus dose ceilings derive from expert opinion rather than from trial data.
Fehlings et al. 2018 [104]	Dystonia	Systematic review and care pathway for dystonia in cerebral palsy, 28 extracted articles, three of them on botulinum toxin.	AMSTAR-2	A priori design and PICO met, a comprehensive librarian-guided search met, duplicate selection and extraction met, evidence graded by the American Academy of Neurology class, protocol registration not reported, publication bias not assessed, pooling items not applicable	Moderate confidence	Rated botulinum toxin level U for inadequate data against level C for intrathecal baclofen and deep brain stimulation, so clinical use rests on expert opinion.

Fifty-three records are listed in Table S1, of which forty-six were appraised with a study-level instrument, four could not be appraised because no full text was obtainable, and three clinical guidance documents were cited as sources of recommendation rather than appraised. The four are those unobtainable studies that are carried out as rows in Table S1, and the wider total, including cited studies that have no row of their own, is given in the outstanding appraisals paragraph below. Twenty-two randomised trials were assessed with RoB 2, one of them using the crossover variant, six nonrandomised studies with ROBINS-I, ten systematic reviews with AMSTAR-2, and eight uncontrolled reports with the Joanna Briggs Institute checklists, seven case series and one case report. No study was excluded on the basis of its appraisal, and every appraised report contributes to the certainty ratings reported in Table 3. Across the appraised evidence, the dominant limitations are unblinded outcome assessment and absence of a control group, with confounding by clinical decision or by historical comparison adding to these in the nonrandomised studies.

Coverage. Appraisal is complete for ten of the seventeen indications. Cerebral palsy of the lower limb has nine of its thirteen primary studies appraised, strabismus four of its five records, functional constipation two of its three sources, congenital muscular torticollis two of its three, primary focal hyperhidrosis two of its three, oesophageal achalasia one of its two, and sialorrhoea four of its five.

One review at high confidence. Bohn 2021 [105] in dystonia meets every AMSTAR-2 critical domain, including a prospectively registered protocol, so the very low certainty for dystonia reflects a weak underlying literature rather than a weak review.

Two reviews at critically low confidence. Friedmacher and Puri 2012 [30] in functional constipation and Mavridi 2024 [127] in chronic migraine each fail three critical domains, in both cases because the included studies were never appraised for quality or risk of bias, so neither review strengthens its indication.

Two reviews at low confidence for one domain only. Gresits 2025 [90] and Rowe and Noonan 2017 [120] fail only the publication bias domain, in each case because there were too few studies to test for it rather than through omission by the review authors.

Table S2. Certainty rating, supporting references, and risk of bias by indication, summarised from Table S1.

This review is a narrative review supported by a structured search and is not a systematic review. Consistent with that design, we appraised the studies on which the certainty rating of each indication depends rather than every reference cited in the manuscript, since a substantial part of the reference list consists of historical, pharmacological, regulatory and guideline sources for which a clinical risk-of-bias instrument is not meaningful. Randomised controlled trials were appraised with the revised Cochrane risk-of-bias tool across the domains of randomisation process, deviations from intended interventions, missing outcome data, measurement of the outcome and selection of the reported result, with an overall judgement of low risk, some concerns, or high risk. Nonrandomised comparative studies and cohorts were appraised with ROBINS-I across the domains of confounding, selection of participants, classification of interventions, deviations from intended interventions, missing data, measurement of outcomes and selection of the reported result, with an overall judgement of low, moderate, serious or critical risk. Case series and uncontrolled reports were appraised with the Joanna Briggs Institute critical appraisal checklist for case series. Systematic reviews and meta-analyses were appraised with AMSTAR-2. Clinical practice guidelines and expert consensus statements are cited as sources of recommendation rather than as primary evidence, so they are not appraised with a study-level instrument, and this is recorded in the source type column. No study was excluded on the basis of its appraisal. Reference numbers correspond to the reference list of the main manuscript. The per-study appraisals underlying each summary are given in Table S1.

**Indication**	**Certainty rating in Table 3**	**Key references**	**Source type**	**Predominant study design**	**Risk of bias summary across the studies for this indication, from Table S1**
Cerebral palsy, lower limb spasticity	High for tone, low for function	Simpson 2008 [9], Delgado 2010 [77], Delgado 2016 [79], Koman 1994 [80], Koman 2000 [81], Baker 2002 [82], Sutherland 1999 [83], Ubhi 2000 [84], Corry 1998 [85], Reddihough 2002 [86], Scholtes 2006 [87], Tedroff 2010 [88], Fehlings 2026 [89], Cosgrove 1994 [91], Graham 2008 [92], a randomised controlled trial finding that botulinum toxin combined with bracing did not prevent progressive hip displacement	Two clinical practice guidelines, [9,77]. Thirteen primary studies, [79–89,91,92], one of which, [86], is a crossover trial. Refs. [91,92] are unappraised.	Randomised double-blind placebo-controlled trials of lower limb injection, one of them a crossover trial, [86], together with one prospective controlled cohort, [89]	Nine of thirteen primary studies appraised, predominantly high-risk driven by unblinded outcome assessment and non-equivalent comparators, two at low risk.
Cerebral palsy, upper limb spasticity	Moderate for tone and goal attainment, low for measured function	Strobl 2015 [78], Gresits 2025 [90], Delgado 2021 [95], Dabrowski 2021 [99], Corry 1997 [100], Russo 2007 [101], Olesch 2010 [102], Kawamura 2007 [103]	One expert consensus statement, [78]. One systematic review with meta-analysis, [90]. Six primary studies, [95,99–103]. All six primary studies are randomised trials, Ref. [100] having been confirmed as randomised from its full text.	Randomised controlled trials of upper limb injection, one of them single-blind and one a dose-comparison trial without a placebo arm, together with one systematic review with meta-analysis and one best practice consensus document	All seven appraisable records were appraised, predominantly high risk driven by unblinded outcome assessment, with Delgado 2021 [95] at low risk.
Functional constipation and internal anal sphincter dysfunction	Low to moderate	Keshtgar 2007 [26], Ahmadi 2013 [29], Friedmacher 2012 [30]	Two randomised controlled trials, [26,29]. One meta-analysis, [30].	Two small randomised trials and one meta-analysis	Two of three sources appraised, some concerns for the trial and critically low confidence for the review, driven by unappraised included studies.
Persistent obstruction after surgery for Hirschsprung disease	Low	Church 2017 [39], Roorda 2019 [40]	One primary retrospective study, [39]. One systematic review with meta-analysis, [40].	One retrospective clinical study and one systematic review with meta-analysis	Both records appraised serious risk driven by confounding and non-independent groups and low confidence for the review lacking a registered protocol.

Chronic anal fissure	Very low	Husberg 2009 [42], Keshtgar 2009 [43]	Two primary studies, [42,43].	Small paediatric series	Both records are appraised as high risk, driven by the absence of a control group, with a historical control comparator in Keshtgar 2009 [43].
Oesophageal achalasia	Low	Hurwitz 2000 [44], Ip 2000 [45]	Two primary studies, [44,45].	Case series	One of two records appraised, high risk driven by the absence of a control group in a retrospective case series of 7 children.
Neurogenic detrusor overactivity	Moderate	Austin 2021 [56]	One primary randomised trial, [56].	One adequately powered phase 3 randomised trial	Single record appraised, some concerns driven by imputation of missing diary data, with no placebo arm since the 50 unit group was the comparator.
Idiopathic overactive bladder	Low	Hoebeke 2006 [67], Ringoir 2020 [68]	Two primary cohort studies, [67,68].	Retrospective and small prospective cohorts	Both records appraised as serious to high risk, driven by absence of a control group.
Detrusor sphincter dyssynergia and dysfunctional voiding	Very low	Steinhardt 1997 [71]	One primary case series, [71].	Small case series	Single record appraised, high risk driven by a case report of one child, so no inference on effect size is possible.
Posterior urethral valve bladder	Low	Mokhless 2014 [76]	One randomised controlled trial, [76].	Randomised comparison of endoscopic injection at the bladder neck against cystourethroscopy alone in 20 boys	Single record appraised, some concerns with serious imprecision driven by unstated randomisation, absent sham injection and a sample of 20.
Sialorrhoea	High	Reid 2008 [108], Berweck 2021 [109], Hung 2021 [110], Bekkers 2019 [113], Alrefai 2009 [114]	Four randomised controlled trials, [108,109,113,114]. One systematic review with meta-analysis, [110].	Randomised placebo-controlled trials of salivary gland injection, together with one randomised comparison against submandibular duct surgery, [113].	Four of five sources appraised. The rating rests on the SIPEXI and Alrefai placebo-controlled trials and the Bekkers randomised comparison against surgery, which favoured duct ligation on effect size.
Congenital muscular torticollis	Very low	Joyce 2005 [116], Sinn 2023 [117], Limpaphayom 2019 [119], a combined protocol study, unappraised	Three primary studies, [116,117,119], of which [119] is unappraised.	Case series and one combined protocol study	Two of three records appraised, serious to high risk driven by the absence of a control group against a favourable natural history.
Strabismus	Low overall, moderate in acute acquired comitant esotropia	Rowe 2017 [120], Wan 2017 [122], Tejedor 1998 [123], Tejedor 1999 [124], Nguyen 2025 [125]	One Cochrane systematic review, [120]. Four primary comparative studies, [122–125], two of them randomised and two retrospective nonrandomised.	One Cochrane systematic review, two randomised trials by Tejedor and Rodriguez comparing injection with reoperation in 47 and in 55 children, and two retrospective nonrandomised comparative studies including a three-year multicentre noninferiority study of 76 children by Nguyen et al.	Four of five records appraised, predominantly high risk driven by unblinded assessment, Wan 2017 [122] serious from confounding, and Nguyen 2025 [125] full text not obtainable.
Dystonia	Very low	Fehlings 2018 [104], Bohn 2021 [105]	Two systematic reviews, [104,105], the second an update with meta-analysis.	Two systematic reviews of pharmacological and neurosurgical interventions, one with meta-analysis	Both reviews appraised moderate and high confidence, and both returned

					inadequate or low-quality evidence for botulinum toxin specifically.
Neonatal brachial plexus palsy	Low	DeMatteo 2006 [130], Chen 2022 [132]	One primary clinical study, [130]. One systematic review with meta-analysis, [132].	One primary clinical study and one systematic review with meta-analysis	Both records are appraised as high risk, driven by the absence of a control group, and low confidence for the review lacking a registered protocol.
Chronic migraine in adolescents	Low	Lindsay 2024 [126], Mavridi 2024 [127]	Two systematic reviews, [126] with meta-analysis and [127] without.	Two systematic reviews of paediatric randomised and observational evidence, one with meta-analysis	Both records appraised low confidence and critically low confidence, driven by absent protocol registration and unappraised included studies.
Primary focal hyperhidrosis	Low to moderate	Kouris 2015 [137], Coutinho dos Santos 2009 [138], Glaser 2015 [139], a multicentre open-label adolescent study, unappraised	Three primary studies, [137–139], of which [139] is unappraised.	One multicentre open-label paediatric study and case series	Two of three records appraised, high risk driven by the absence of a control group in an uncontrolled series of 35 and nine patients.

References

9. Simpson, D.M.; Gracies, J.M.; Graham, H.K.; Miyasaki, J.M.; Naumann, M.; Russman, B.; Simpson, L.L.; So, Y. Assessment: Botulinum neurotoxin for the treatment of spasticity (an evidence-based review): Report of the Therapeutics and Technology Assessment Subcommittee of the American Academy of Neurology. Neurology 2008, 70, 1691–1698.

26. Keshtgar, A.S.; Ward, H.C.; Sanei, A.; Clayden, G.S. Botulinum Toxin, a New Treatment Modality for Chronic Idiopathic Constipation in Children: Long-Term Follow-Up of a Double-Blind Randomized Trial. J. Pediatr. Surg. 2007, 42, 672–680.

29. Ahmadi, J.; Azary, S.; Ashjaei, B.; Paragomi, P.; Khalifeh-Soltani, A. Intrasphincteric Botulinum Toxin Injection in Treatment of Chronic Idiopathic Constipation in Children. Iran. J. Pediatr. 2013, 23, 574–578.

30. Friedmacher, F.; Puri, P. Comparison of Posterior Internal Anal Sphincter Myectomy and Intrasphincteric Botulinum Toxin Injection for Treatment of Internal Anal Sphincter Achalasia: A Meta-Analysis. Pediatr. Surg. Int. 2012, 28, 765–771.

39. Church, J.T.; Gadepalli, S.K.; Talishinsky, T.; Teitelbaum, D.H.; Jarboe, M.D. Ultrasound-Guided Intrasphincteric Botulinum Toxin Injection Relieves Obstructive Defecation Due to Hirschsprung's Disease and Internal Anal Sphincter Achalasia. J. Pediatr. Surg. 2017, 52, 74–78.

40. Roorda, D.; Abeln, Z.A.; Oosterlaan, J.; van Heurn, L.W.; Derikx, J.P. Botulinum Toxin Injections after Surgery for Hirschsprung Disease: Systematic Review and Meta-Analysis. World J. Gastroenterol. 2019, 25, 3268–3280.

42. Husberg, B.; Malmborg, P.; Strigård, K. Treatment with Botulinum Toxin in Children with Chronic Anal Fissure. Eur. J. Pediatr. Surg. 2009, 19, 290–292.

43. Keshtgar, A.S.; Ward, H.C.; Clayden, G.S. Transcutaneous Needle-Free Injection of Botulinum Toxin: A Novel Treatment of Childhood Constipation and Anal Fissure. J. Pediatr. Surg. 2009, 44, 1791–1798.

44. Hurwitz, M.; Bahar, R.J.; Ament, M.E.; Tolia, V.; Molleston, J.; Reinstein, L.J.; Walton, J.M.; Erhart, N.; Wasserman, D.; Justinich, C.; et al. Evaluation of the Use of Botulinum Toxin in Children with Achalasia. J. Pediatr. Gastroenterol. Nutr. 2000, 30, 509–514.

45. Ip, K.S.; Cameron, D.J.; Catto-Smith, A.G.; Hardikar, W. Botulinum Toxin for Achalasia in Children. J. Gastroenterol. Hepatol. 2000, 15, 1100–1104.

56. Austin, P.F.; Franco, I.; Dobremez, E.; Kroll, P.; Titanji, W.; Geib, T.; Jenkins, B.; Hoebeke, P.B. OnabotulinumtoxinA for the Treatment of Neurogenic Detrusor Overactivity in Children. Neurourol. Urodyn. 2021, 40, 493–501.

67. Hoebeke, P.; De Caestecker, K.; Vande Walle, J.; Dehoorne, J.; Raes, A.; Verleyen, P.; Van Laecke, E. The Effect of Botulinum-A Toxin in Incontinent Children with Therapy Resistant Overactive Detrusor. J. Urol. 2006, 176, 328–330.

68. Ringoir, A.; Dhondt, B.; De Bleser, E.; Van Laecke, E.; Everaert, K.; Groen, L.A.; Hoebeke, P.; Spinoit, A.F. Intradetrusor Onabotulinum-A Toxin Injections in Children with Therapy-Resistant Idiopathic Detrusor Overactivity. A Retrospective Study. J. Pediatr. Urol. 2020, 16, 181.e1–181.e8.

71. Steinhardt, G.F.; Naseer, S.; Cruz, O.A. Botulinum Toxin: Novel Treatment for Dramatic Urethral Dilatation Associated with Dysfunctional Voiding. J. Urol. 1997, 158, 190–191.

76. Mokhless, I.; Zahran, A.R.; Saad, A.; Yehia, M.; Youssif, M.E. Effect of Botox Injection at the Bladder Neck in Boys with Bladder Dysfunction after Valve Ablation. J. Pediatr. Urol. 2014, 10, 899–904.

77. Delgado, M.R.; Hirtz, D.; Aisen, M.; Ashwal, S.; Fehlings, D.L.; McLaughlin, J.; Morrison, L.A.; Shrader, M.W.; Tilton, A.; Vargus-Adams, J. Practice Parameter: Pharmacologic Treatment of Spasticity in Children and Adolescents with Cerebral Palsy (an Evidence-Based Review): Report of the Quality Standards Subcommittee of the American Academy of Neurology and the Practice Committee of the Child Neurology Society. Neurology 2010, 74, 336–343.

78. Strobl, W.; Theologis, T.; Brunner, R.; Kocer, S.; Viehweger, E.; Pascual-Pascual, I.; Placzek, R. Best Clinical Practice in Botulinum Toxin Treatment for Children with Cerebral Palsy. Toxins 2015, 7, 1629–1648.

79. Delgado, M.R.; Tilton, A.; Russman, B.; Benavides, O.; Bonikowski, M.; Carranza, J.; Dabrowski, E.; Dursun, N.; Gormley, M.; Jozwiak, M.; et al. AbobotulinumtoxinA for Equinus Foot Deformity in Cerebral Palsy: A Randomized Controlled Trial. Pediatrics 2016, 137, e20152830.

80. Koman, L.A.; Mooney, J.F., 3rd; Smith, B.; Goodman, A.; Mulvaney, T. Management of Spasticity in Cerebral Palsy with Botulinum-A Toxin: Report of a Preliminary, Randomized, Double-Blind Trial. J. Pediatr. Orthop. 1994, 14, 299–303.

81. Koman, L.A.; Mooney, J.F., 3rd; Smith, B.P.; Walker, F.; Leon, J.M.; BOTOX Study Group. Botulinum Toxin Type A Neuromuscular Blockade in the Treatment of Lower Extremity Spasticity in Cerebral Palsy: A Randomized, Double-Blind, Placebo-Controlled Trial. J. Pediatr. Orthop. 2000, 20, 108–115.

82. Baker, R.; Jasinski, M.; Maciag-Tymecka, I.; Michalowska-Mrozek, J.; Bonikowski, M.; Carr, L.; MacLean, J.; Lin, J.P.; Lynch, B.; Theologis, T.; et al. Botulinum Toxin Treatment of Spasticity in Diplegic Cerebral Palsy: A Randomized, Double-Blind, Placebo-Controlled, Dose-Ranging Study. Dev. Med. Child Neurol. 2002, 44, 666–675.

83. Sutherland, D.H.; Kaufman, K.R.; Wyatt, M.P.; Chambers, H.G.; Mubarak, S.J. Double-Blind Study of Botulinum A Toxin Injections into the Gastrocnemius Muscle in Patients with Cerebral Palsy. Gait Posture 1999, 10, 1–9.

84. Ubhi, T.; Bhakta, B.B.; Ives, H.L.; Allgar, V.; Roussounis, S.H. Randomised Double Blind Placebo Controlled Trial of the Effect of Botulinum Toxin on Walking in Cerebral Palsy. Arch. Dis. Child. 2000, 83, 481–487.

85. Corry, I.S.; Cosgrove, A.P.; Duffy, C.M.; McNeill, S.; Taylor, T.C.; Graham, H.K. Botulinum Toxin A Compared with Stretching Casts in the Treatment of Spastic Equinus: A Randomised Prospective Trial. J. Pediatr. Orthop. 1998, 18, 304–311.

86. Reddihough, D.S.; King, J.A.; Coleman, G.J.; Fosang, A.; McCoy, A.T.; Thomason, P.; Graham, H.K. Functional Outcome of Botulinum Toxin A Injections to the Lower Limbs in Cerebral Palsy. Dev. Med. Child Neurol. 2002, 44, 820–827.

87. Scholtes, V.A.; Dallmeijer, A.J.; Knol, D.L.; Speth, L.A.; Maathuis, C.G.; Jongerius, P.H.; Becher, J.G. The Combined Effect of Lower-Limb Multilevel Botulinum Toxin Type A and Comprehensive Rehabilitation on Mobility in Children with Cerebral Palsy: A Randomized Clinical Trial. Arch. Phys. Med. Rehabil. 2006, 87, 1551–1558.

88. Tedroff, K.; Löwing, K.; Haglund-Åkerlind, Y.; Gutierrez-Farewik, E.; Forssberg, H. Botulinum toxin A treatment in toddlers with cerebral palsy. Acta Paediatr. 2010, 99, 1156–1162.

89. Fehlings, D.; Bohn, E.; Switzer, L.; Goldsmith, C.H.; Narayanan, U.; Rosenbaum, P.L.; Wright, F.V.; Foster, G.; CP-BoNT-A Study Group. Three-Year Outcomes of Repeated Botulinum Neurotoxin A Injections to the Lower Extremities in Young Children with Spastic Cerebral Palsy in GMFCS Levels I to III. Dev. Med. Child Neurol. 2026, 68, 792–799.

90. Gresits, O.Z.; Vezér, M.; Engh, M.A.; Szabó, L.; Molnár, Z.; Hegyi, P.; Terebessy, T. Limited Evidence of Functional Benefit After Upper Limb Botulinum Toxin Treatment in Children With Cerebral Palsy. Systematic Review and Meta-analysis. Am. J. Phys. Med. Rehabil. 2025, 104, 108–116.

91. Cosgrove, A.P.; Corry, I.S.; Graham, H.K. Botulinum Toxin in the Management of the Lower Limb in Cerebral Palsy. Dev. Med. Child Neurol. 1994, 36, 386–396.

92. Graham, H.K.; Boyd, R.; Carlin, J.B.; Dobson, F.; Lowe, K.; Nattrass, G.; Thomason, P.; Wolfe, R.; Reddihough, D. Does Botulinum Toxin A Combined with Bracing Prevent Hip Displacement in Children with Cerebral Palsy and 'Hips at Risk'? A Randomized, Controlled Trial. J. Bone Jt. Surg. Am. 2008, 90, 23–33.

95. Delgado, M.R.; Tilton, A.; Carranza-Del Río, J.; Dursun, N.; Bonikowski, M.; Aydin, R.; Maciag-Tymecka, I.; Oleszek, J.; Dabrowski, E.; Grandoulier, A.S.; et al. Efficacy and Safety of AbobotulinumtoxinA for Upper Limb Spasticity in Children with Cerebral Palsy: A Randomized Repeat-Treatment Study. Dev. Med. Child Neurol. 2021, 63, 592–600.

99. Dabrowski, E.; Chambers, H.G.; Gaebler-Spira, D.; Banach, M.; Kaňovský, P.; Dersch, H.; Hanschmann, A.; Vilain, C.; Pulte, I.; Althaus, M.; et al. IncobotulinumtoxinA Efficacy/Safety in Upper-Limb Spasticity in Pediatric Cerebral Palsy: Randomized Controlled Trial. Pediatr. Neurol. 2021, 123, 10–20.

100. Corry, I.S.; Cosgrove, A.P.; Walsh, E.G.; McClean, D.; Graham, H.K. Botulinum Toxin A in the Hemiplegic Upper Limb: A Double-Blind Trial. Dev. Med. Child Neurol. 1997, 39, 185–193.

101. Russo, R.N.; Crotty, M.; Miller, M.D.; Murchland, S.; Flett, P.; Haan, E. Upper-Limb Botulinum Toxin A Injection and Occupational Therapy in Children with Hemiplegic Cerebral Palsy Identified from a Population Register: A Single-Blind, Randomized, Controlled Trial. Pediatrics 2007, 119, e1149–e1158.

102. Olesch, C.A.; Greaves, S.; Imms, C.; Reid, S.M.; Graham, H.K. Repeat Botulinum Toxin-A Injections in the Upper Limb of Children with Hemiplegia: A Randomized Controlled Trial. Dev. Med. Child Neurol. 2010, 52, 79–86.

103. Kawamura, A.; Campbell, K.; Lam-Damji, S.; Fehlings, D. A Randomized Controlled Trial Comparing Botulinum Toxin A Dosage in the Upper Extremity of Children with Spasticity. Dev. Med. Child Neurol. 2007, 49, 331–337.

104. Fehlings, D.; Brown, L.; Harvey, A.; Himmelmann, K.; Lin, J.P.; Macintosh, A.; Mink, J.W.; Monbaliu, E.; Rice, J.; Silver, J.; et al. Pharmacological and Neurosurgical Interventions for Managing Dystonia in Cerebral Palsy: A Systematic Review. Dev. Med. Child Neurol. 2018, 60, 356–366.

105. Bohn, E.; Goren, K.; Switzer, L.; Falck-Ytter, Y.; Fehlings, D. Pharmacological and Neurosurgical Interventions for Individuals with Cerebral Palsy and Dystonia: A Systematic Review Update and Meta-Analysis. Dev. Med. Child Neurol. 2021, 63, 1038–1050.

108. Reid, S.M.; Johnstone, B.R.; Westbury, C.; Rawicki, B.; Reddihough, D.S. Randomized Trial of Botulinum Toxin Injections into the Salivary Glands to Reduce Drooling in Children with Neurological Disorders. Dev. Med. Child Neurol. 2008, 50, 123–128.

109. Berweck, S.; Bonikowski, M.; Kim, H.; Althaus, M.; Flatau-Baqué, B.; Mueller, D.; Banach, M.D. Placebo-Controlled Clinical Trial of IncobotulinumtoxinA for Sialorrhea in Children: SIPEXI. Neurology 2021, 97, e1425–e1436.

110. Hung, S.A.; Liao, C.L.; Lin, W.P.; Hsu, J.C.; Guo, Y.H.; Lin, Y.C. Botulinum Toxin Injections for Treatment of Drooling in Children with Cerebral Palsy: A Systematic Review and Meta-Analysis. Children 2021, 8, 1089.

113. Bekkers, S.; Delsing, C.P.; Kok, S.E.; van Hulst, K.; Erasmus, C.E.; Scheffer, A.R.T.; van den Hoogen, F.J.A. Randomized Controlled Trial Comparing Botulinum vs Surgery for Drooling in Neurodisabilities. Neurology 2019, 92, e1195–e1204.

114. Alrefai, A.H.; Aburahma, S.K.; Khader, Y.S. Treatment of Sialorrhea in Children with Cerebral Palsy: A Double-Blind Placebo-Controlled Trial. Clin. Neurol. Neurosurg. 2009, 111, 79–82.

116. Joyce, M.B.; de Chalain, T.M. Treatment of Recalcitrant Idiopathic Muscular Torticollis in Infants with Botulinum Toxin Type A. J. Craniofac. Surg. 2005, 16, 321–327.

117. Sinn, C.N.; Rinaldi, R.J.; McLaughlin, M.J. Botulinum Toxin Type A Outcomes in Infants with Refractory Congenital Muscular Torticollis. J. Pediatr. Rehabil. Med. 2023, 16, 539–552.

119. Limpaphayom, N.; Kohan, E.; Huser, A.; Michalska-Flynn, M.; Stewart, S.; Dobbs, M.B. Use of Combined Botulinum Toxin and Physical Therapy for Treatment Resistant Congenital Muscular Torticollis. J. Pediatr. Orthop. 2019, 39, e343–e348.

120. Rowe, F.J.; Noonan, C.P. Botulinum Toxin for the Treatment of Strabismus. Cochrane Database Syst. Rev. 2017, 3, CD006499.

122. Wan, M.J.; Mantagos, I.S.; Shah, A.S.; Kazlas, M.; Hunter, D.G. Comparison of Botulinum Toxin with Surgery for the Treatment of Acute-Onset Comitant Esotropia in Children. Am. J. Ophthalmol. 2017, 176, 33–39.

123. Tejedor, J.; Rodríguez, J.M. Retreatment of Children after Surgery for Acquired Esotropia: Reoperation versus Botulinum Toxin. Br. J. Ophthalmol. 1998, 82, 110–114.

124. Tejedor, J.; Rodríguez, J.M. Early Retreatment of Infantile Esotropia: Comparison of Reoperation and Botulinum Toxin. Br. J. Ophthalmol. 1999, 83, 783–787.

125. Nguyen, M.T.B.; Cheung, C.S.Y.; Hunter, D.G.; Wan, M.J.; Gise, R. Three-Year Outcomes of Botulinum Toxin versus Strabismus Surgery for the Treatment of Acute Acquired Comitant Esotropia in Children. Am. J. Ophthalmol. 2025, 272, 1–7.

126. Lindsay, R.; Kalifa, A.; Kuziek, J.; Kabbouche, M.; Hershey, A.D.; Orr, S.L. The Safety and Efficacy of OnabotulinumtoxinA Injections for Children and Adolescents with Chronic Migraine: A Systematic Review and Meta-Analysis. Headache 2024, 64, 1200–1216.

127. Mavridi, A.; Redmond, A.; Archontakis-Barakakis, P.; Bogdanova-Mihaylova, P.; Deligianni, C.I.; Mitsikostas, D.D.; Mavridis, T. OnabotulinumtoxinA in the Prevention of Migraine in Pediatric Population: A Systematic Review. Toxins 2024, 16, 295.

130. DeMatteo, C.; Bain, J.R.; Galea, V.; Gjertsen, D. Botulinum Toxin as an Adjunct to Motor Learning Therapy and Surgery for Obstetrical Brachial Plexus Injury. Dev. Med. Child Neurol. 2006, 48, 245–252.

132. Chen, T.Y.; Su, Y.C.; Lin, Y.C.; Guo, Y.H. The Effectiveness and Safety of Botulinum Neurotoxin in Obstetric Brachial Plexus Injury: A Systematic Review and Meta-Analysis. Healthcare 2022, 10, 2419.

137. Kouris, A.; Armyra, K.; Stefanaki, C.; Christodoulou, C.; Karimali, P.; Kontochristopoulos, G. Quality of Life and Social Isolation in Greek Adolescents with Primary Focal Hyperhidrosis Treated with Botulinum Toxin Type A: A Case Series. Pediatr. Dermatol. 2015, 32, 226–230.

138. Coutinho dos Santos, L.H.; Gomes, A.M.; Giraldi, S.; Abagge, K.T.; Marinoni, L.P. Palmar Hyperhidrosis: Long-Term Follow-Up of Nine Children and Adolescents Treated with Botulinum Toxin Type A. Pediatr. Dermatol. 2009, 26, 439–444.

139. Glaser, D.A.; Pariser, D.M.; Hebert, A.A.; Landells, I.; Somogyi, C.; Weng, E.; Beddingfield, F. A Prospective, Nonrandomized, Open-Label Study of the Efficacy and Safety of OnabotulinumtoxinA in Adolescents with Primary Axillary Hyperhidrosis. Pediatr. Dermatol. 2015, 32, 609–617.
